# Conserved use of tetraspanin CD9 as an entry receptor by rhabdoviruses spanning multiple genera

**DOI:** 10.1073/pnas.2530369123

**Published:** 2026-03-11

**Authors:** Wanwan Zhang, Bingbing Sun, Hao Huang, Junyu Chen, Ying’an Liang, Wenxi Li, Qinyu Peng, Ping Zhang, Zhipeng Zhan, Xiaogang Yang, Lan Yao, Huiquan Chen, Eran Bacharach, Haifeng Li, Changjun Guo, Meisheng Yi, Kuntong Jia

**Affiliations:** ^a^School of Marine Sciences, Sun Yat-sen University, Zhuhai 519082, China; ^b^Guangdong Provincial Key Laboratory of Marine Resources and Coastal Engineering, Zhuhai 519082, China; ^c^Department of Immunology and Microbiology, Zhongshan School of Medicine, Sun Yat-sen University, Guangzhou 510080, China; ^d^The Shmunis School of Biomedicine and Cancer Research, George S. Wise Faculty of Life Sciences, Tel Aviv University, Tel Aviv 6997801, Israel; ^e^State Key Laboratory for Biocontrol, School of Life Sciences, Sun Yat-sen University, Guangzhou 510275, China

**Keywords:** CD9, rhabdoviruses, VHSV, VSV, nitazoxanide

## Abstract

Rhabdoviruses exhibit a broad host range, yet the cellular receptors underlying their cross-species tropism remain poorly defined. Here, we identified tetraspanin CD9 as a conserved functional entry receptor for diverse rhabdoviruses across genera, including viral hemorrhagic septicemia virus (VHSV) (*Novirhabdovirus*), *Siniperca chuatsi* rhabdovirus (SCRV) (*Siniperhavirus*), and vesicular stomatitis virus (VSV) (*Vesiculovirus*). We demonstrated that the domain IV of VHSV glycoprotein G directly interacted with the large extracellular loop domain of *Lateolabrax japonicus* CD9 (LjCD9). CD9 knockout, CD9 protein, or CD9 antibody significantly reduced VHSV infection in vitro and CD9 knockout zebrafish, while HEK293T cells, which are nonsusceptible but permissive to VHSV, become susceptible when expressing LjCD9, suggesting that LjCD9 is an entry receptor for VHSV. We further confirmed that LjCD9 functions as a functional receptor of SCRV. Importantly, the human CD9 orthologue can serve as a receptor of VSV. Our findings also revealed that LjCD9 mediated VHSV entry via clathrin- and caveolae-mediated endocytosis. Notably, nitazoxanide (NTZ) was identified as a broad-spectrum inhibitor of VHSV, SCRV, and VSV likely by interfering with the G protein–CD9 interaction. This study establishes CD9 as a cross-species receptor for rhabdoviruses and highlights NTZ as a promising broad-spectrum antiviral agent.

## Significance

Rhabdoviruses infect a wide range of hosts, but the cellular receptors enabling this cross-species infection were unclear. We identified the tetraspanin protein cluster and differentiation 9 (CD9) as a conserved functional entry receptor for diverse rhabdoviruses, including viral hemorrhagic septicemia virus (VHSV), *Siniperca chuatsi* rhabdovirus (SCRV), and vesicular stomatitis virus (VSV). Our work shows that CD9 directly interacts with viral glycoproteins to mediate virus entry through specific endocytic pathways. Importantly, we found that nitazoxanide (NTZ), an Food and Drug Administration (FDA)-approved antiparasitic agent, is currently being repurposed and evaluated as an antiviral candidate against multiple rhabdoviruses. These findings reveal a shared mechanism of rhabdovirus infection across species and suggest a potential broad-spectrum antiviral strategy.

Rhabdoviruses constitute a diverse family of enveloped, negative-sense RNA viruses capable of infecting a wide range of hosts, including mammals, fish, insects, and plants, posing significant threats to human health, veterinary medicine, agriculture, and global aquaculture (1–3). Currently, 56 genera encompassing 434 species have been classified by the International Committee on Taxonomy of Viruses (4). Notable members such as vesicular stomatitis virus (VSV) and viral hemorrhagic septicemia virus (VHSV) cause highly contagious diseases in livestock and aquatic species, with outbreaks leading to devastating economic losses in aquaculture and animal husbandry worldwide (5). Despite decades of research, effective vaccines and targeted therapeutics are lacking for many rhabdoviral diseases; control measures rely heavily on quarantine and culling, which are inadequate to curb transboundary spread (6, 7). The expanding host range and the lack of specific interventions make rhabdovirus infections an ongoing and escalating challenge to both animal health and the aquaculture industry (8–10).

Host cell receptors are fundamental determinants of viral tropism, host range, and cross-species transmission, serving as critical gatekeepers for viral entry and infection (11, 12). For many emerging and economically significant viruses, including members of the family Rhabdoviridae, the molecular identity of entry receptors remains incompletely understood (13). Despite their diversity, all rhabdoviruses rely on viral glycoprotein G to mediate attachment and entry, typically via receptor-mediated endocytosis (14–16). However, the repertoire of host receptors exploited by rhabdoviruses is minimal and often species- or strain-specific (17, 18). The receptors or putative receptors that have been identified for VSV and rabies virus (RABV) are not used by other rhabdoviruses, even from the same genus. For example, lyssaviruses such as Mokola virus, Duvenhage virus, or European bat lyssavirus type 1 do not bind to p75 neurotrophin receptor, a receptor for RABV (19). Similarly, Chandipura virus glycoprotein G does not bind low-density lipoprotein receptor (LDLR) (20), whose family members have been shown to function as VSV receptors in mammals (18). Fish rhabdoviruses, such as VHSV and *Siniperca chuatsi* rhabdovirus (SCRV), represent a less understood group of viruses where the entry mechanisms remain elusive. A few candidate receptor molecules, including fibronectin (21) and phosphatidylserine (22, 23), have been implicated, but the precise receptors for these viruses remain unclear. Importantly, although rhabdoviruses from different genera (e.g., *Novirhabdovirus*, *Siniperhavirus*, *Novirhabdovirus*) can infect fish, there is no clear evidence supporting the existence of a conserved, cross-species receptor that could explain the broad host range of these viruses.

Tetraspanin has emerged as a potential entry factor for a variety of viruses, highlighting its relevance in viral pathogenesis and tropism (24–27). Members of tetraspanin family like CD9 have been implicated in facilitating entry of multiple viral pathogens through modulation of receptor clustering and membrane fusion (28–31). For example, CD9 facilitates MERS-coronavirus (CoV) entry by binding to its primary viral receptor, DPP4, and the CoV-activating protease TMPRSS2 (32). In fish, VHSV infection upregulates CD9 expression in rainbow trout, suggesting a potential role in viral entry (33). However, it remains unclear whether CD9 functions as a receptor for fish rhabdoviruses or a conserved receptor for diverse rhabdoviruses, especially across genera.

Here, we report the identification of tetraspanin CD9 as a conserved functional entry receptor for multiple rhabdoviruses, including VHSV (*Novirhabdovirus*), VSV (*Vesiculovirus*), and SCRV (*Siniperhavirus*). Critically, we identified nitazoxanide (NTZ), a small-molecule inhibitor targeting domain IV of the G protein, as an effective agent capable of suppressing VHSV, SCRV, and VSV infections. Our findings reveal CD9 as a previously unrecognized, cross-species entry receptor for rhabdoviruses, thereby providing critical mechanistic insights into rhabdovirus pathogenesis and a potential target for broad-spectrum antiviral therapy.

## Results

### LjCD9 Is Required for Efficient VHSV Entry.

Our prior transcriptomic analysis linked CD9 from sea perch (*Lateolabrax japonicus*; LjCD9) to VHSV infection. To further investigate this relationship, we first examined *LjCD9* mRNA expression in various tissues and cells of sea perch. We found that *LjCD9* expression significantly increased in liver, kidney, gill, eye, and brain tissues after VHSV infection (*SI Appendix*, Fig. S1*A*). Additionally, *LjCD9* mRNA was upregulated in the brain and liver tissues of VHSV-infected fish at different time intervals, consistent with the elevated levels of *LjCD9* expression in *L. japonicus* brain cells (LJB) cells at 2- and 4-hours postinfection (hpi) (*SI Appendix*, Fig. S1*B*). Furthermore, LjCD9 overexpression significantly increased VHSV infection, as indicated by elevated *RNA levels of the viral G, M, and N genes* (*SI Appendix*, Fig. S1 *C*–*E*), as well as increased viral titer in the supernatant at 24 hpi (*SI Appendix*, Fig. S1*F*). Especially, ectopic expression of LjCD9 facilitated VHSV entry, as suggested by the upregulation of *G* and *N* (*SI Appendix*, Fig. S1 *G* and *H*) mRNA expression at 4 hpi and G protein expression at 24 hpi in the LjCD9 overexpression group compared to the empty vector control group. Conversely LjCD9 knockdown exerted the opposite effect relative to the negative control (NC) group (*SI Appendix*, Fig. S1*I*). These findings indicate LjCD9 plays a key role in the early steps of VHSV infection.

### LjCD9 Directly Interacts with the VHSV G Protein.

**Fig. 1.**
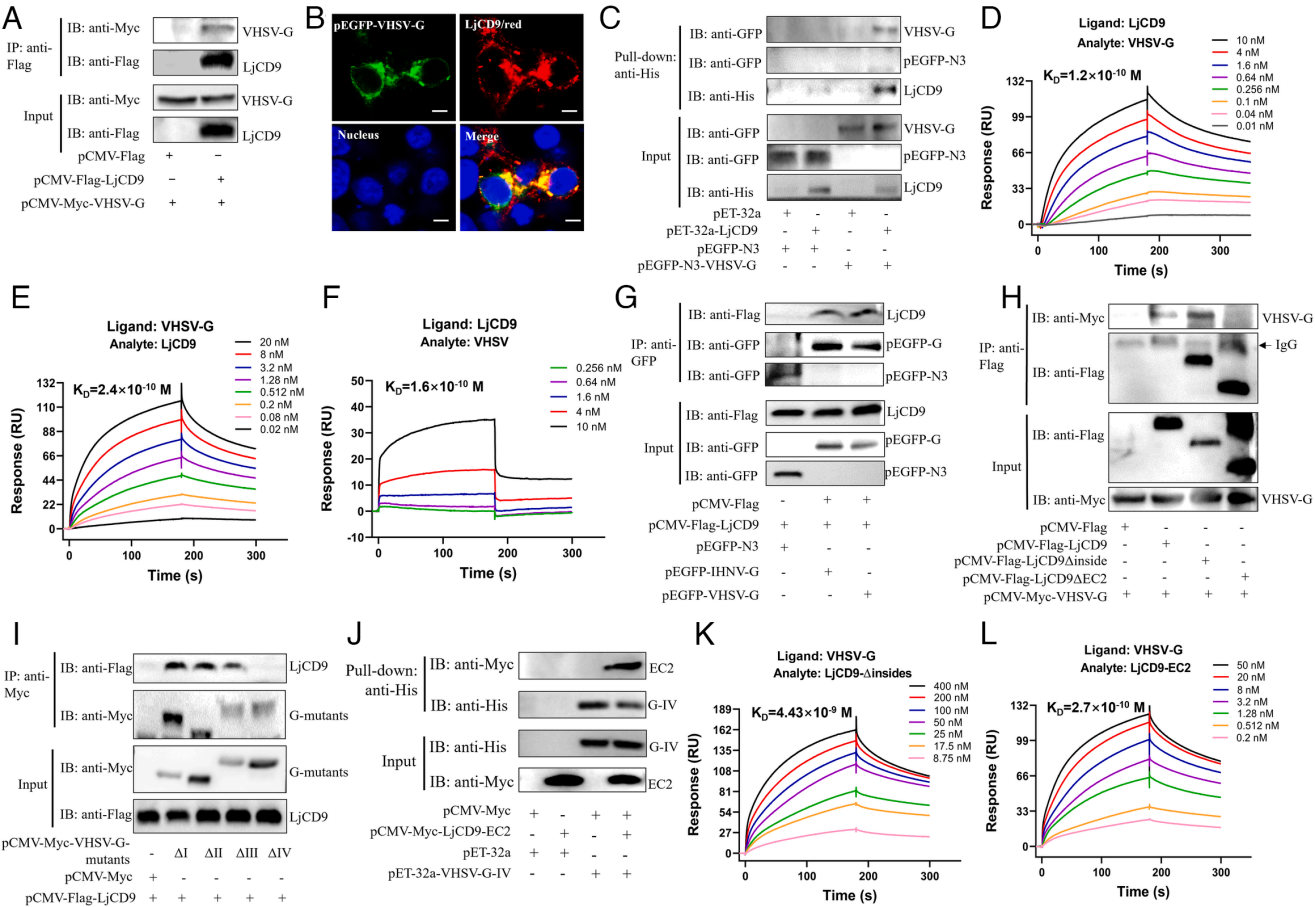
Direct interaction between LjCD9 EC2 domain and domain IV of VHSV G protein. (*A*) Co-IP of LjCD9 and VHSV G protein. HEK293T cells were transfected with various plasmids as indicated. The cell lysates were then subjected to immunoprecipitation using anti-Flag magnetic beads followed by Western blot analysis. (*B*) Immunofluorescence staining of LjCD9 and VHSV G in HEK293T cells cotransfected with *Flag-LjCD9* and *pEGFP-VHSV-G* plasmids. LjCD9 (Red) was detected by anti-Flag antibody, and the nucleus was stained by DAPI, Bar = 10 μm. (*C*) LjCD9 pulls down VHSV G. The lysates of HEK293T cells transfected with indicated plasmids were pulled down with purified His-LjCD9 or His proteins using anti-His magnetic beads. The proteins directly bound to LjCD9 were immunoblotted with anti-His and anti-GFP antibodies, respectively. (*D*) Surface plasmon resonance (SPR) analysis of VHSV G binding to immobilized LjCD9. LjCD9 protein was immobilized on a CM5 sensor chip, and VHSV G protein was passed at the indicated concentrations. (*E*) SPR analysis of LjCD9 binding to immobilized VHSV G protein. VHSV G protein was immobilized on a CM5 sensor chip, and LjCD9 protein was passed at the indicated concentrations. (*F*) SPR analysis of VHSV binding to immobilized LjCD9 protein. LjCD9 protein was immobilized on a CM5 sensor chip, and VHSV (10^7^ pfu/mL) suspended in PBS was passed at the indicated concentrations. (*G*) Co-IP of LjCD9 with IHNV G or VHSV G. HEK293T cells transfected with Flag-LjCD9 and GFP-IHNV G/VHSV G plasmids were immunoprecipitated with anti-GFP beads to compare binding specificity. (*H*) Co-IP of VHSV G with LjCD9 truncated mutants. A series of LjCD9 mutants and VHSV G were cotransfected into HEK293T cells for 24 h and subjected to immunoprecipitation with the anti-Flag magnetic beads, followed by immunoblotting analysis. (*I*) Co-IP of LjCD9 with different domains deletion mutants of VHSV G (Δdomain I–IV). Cell lysates of HEK293T cells cotransfected with pCMV-Flag-LjCD9 and a series of VHSV G mutants for 24 h were immunoprecipitated with the anti-Myc magnetic beads, followed by immunoblotting analysis for the complex with the indicated antibodies. (*J*) The EC2 domain of LjCD9 pulls down VHSV G protein domain IV. The lysates of HEK293T cells transfected with LjCD9-EC2 domain plasmids were pulled down with purified His-VHSV-G protein domain IV or His proteins using anti-His magnetic beads, and immunoblotted with anti-His and anti-Myc antibodies, respectively. (*K* and *L*) SPR analysis of mutant LjCD9 binding to immobilized VHSV G protein. VHSV G protein was immobilized on a CM5 sensor chip, and LjCD9 protein lacking insides motifs (CD9-Δinsides) (*K*) and LjCD9 EC2 domain alone (LjCD9-EC2) (*L*) were passed at the indicated concentrations.

The G protein has been reported to play a vital role in rhabdovirus entry into host cells through binding to host receptors (13, 20). We therefore investigated whether LjCD9 interacts with the VHSV G protein. The results of the coimmunoprecipitation (Co-IP) assay showed that the VHSV G and LjCD9 proteins coimmunoprecipitated with each other (Fig. 1*A* and *SI Appendix*, Fig. S2*A*). The colocalization of VHSV G and LjCD9 on the cell surface further supported their interaction (Fig. 1*B*). Furthermore, an in vitro pull-down assay using purified His-LjCD9 protein indicated a direct interaction between LjCD9 and VHSV G protein (Fig. 1*C*). Surface plasmon resonance (SPR) analysis corroborated the direct interaction between VHSV G and LjCD9 protein. Dose–response binding of VHSV G protein to immobilized LjCD9 protein yielded an association rate constant (Ka) of 1.58 × 10^7^ M^−1^ s^−1^, a dissociation rate constant (Kd) of 1.90 × 10^−3^ s^−1^, and an equilibrium dissociation constant (K_D_) of 1.2 × 10^−10^ M (0.12 nM) (Fig. 1*D* and *SI Appendix*, Fig. S2*B*). The binding of LjCD9 protein to immobilized VHSV G protein revealed the Ka, Kd, and K_D_ for their interactions were 8.28 × 10^6^ M^−1^ s^−1^, 1.97 × 10^−3^ s^−1^, and 2.4 ×10^−10^ M (0.24 nM), respectively (Fig. 1*E* and *SI Appendix*, Fig. S2*C*). The binding of VHSV particles to immobilized LjCD9 protein was detected and revealed a K_D_ value of 1.6 ×10^−10^ M (0.16 nM) (Fig. 1*F*), indicating a very high avidity. Moreover, we found that CD9 from largemouth bass (*Micropterus salmoides*) (MsCD9) could bind to the VHSV G protein (*SI Appendix*, Fig. S2*D*). The G protein of infectious hematopoietic necrosis virus (IHNV), a novirhabdovirus in the same genus as VHSV, also interacted with LjCD9 (Fig. 1*G*), suggesting that the interaction between fish CD9 and fish rhabdoviruses G protein is conserved.

To identify the specific domain involved in the interaction between VHSV G and LjCD9, truncations of VHSV G and LjCD9 were generated for Co-IP analysis. The results revealed that truncated LjCD9 lacking inside motifs (Δinside) retained some ability to bind VHSV G, whereas LjCD9 lacking the extracellular loop 2 motif (EC2) (ΔEC2) lost its association ability (Fig. 1*H* anf *SI Appendix*, Fig. S2*E*). Furthermore, the domain IV of VHSV G is found crucial for the interaction, as the truncated Δdomain IV protein failed to interact with LjCD9, whereas the truncated Δdomains I, II, and III retained the ability to interact with LjCD9 (Fig. 1*I* and *SI Appendix*, Fig. S2*E*). Pull-down assays further verified the direct interaction between VHSV G protein domain IV and LjCD9-EC2 domain (Fig. 1*J*). The interactions between VHSV G and LjCD9 truncated mutants were also investigated using SPR. The K_D_ value for dose–response binding of truncated LjCD9, lacking inside motifs, to immobilized VHSV G was approximately 4.43 ×10^−9^ M (4.43 nM) (Fig. 1*K*). In contrast, no avidity was measured between LjCD9-ΔEC2 and immobilized VHSV G protein (*SI Appendix*, Fig. S2*F*). When only the EC2 motif of LjCD9 was detected for its avidity with VHSV G, the K_D_ reached approximately 2.7 ×10^−10^ M (0.27 nM) (Fig. 1*L*). These results suggested that the domain IV of VHSV G protein binds to the EC2 motif of LjCD9.

### LjCD9 Serves as a Receptor to Facilitate VHSV Entry.

**Fig. 2.**
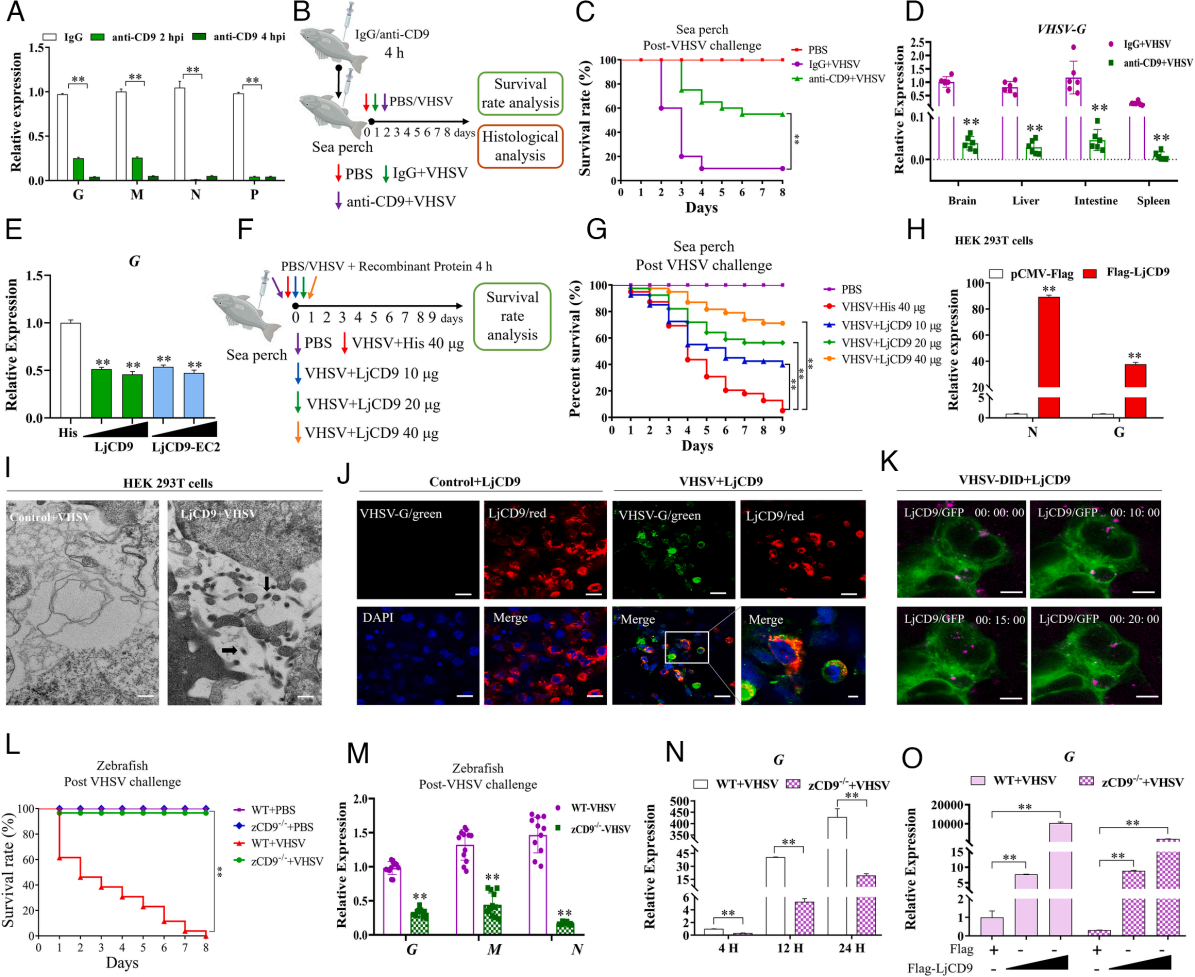
Validation of LjCD9 as a potential host receptor for VHSV. (*A*) RT-qPCR analysis of VHSV *G*, *M*, *N*, and *P* mRNA in LJB cells pretreated with anti-human CD9 antibody (1:50) for 4 h, followed by VHSV infection at 28 °C for 2 or 4 h. (*B*–*D*) Effect of anti-CD9 antibody on VHSV infection in vivo. Sea perch were intraperitoneally injected with 10 μg anti-CD9 antibody in 30 μL phosphate-buffered saline (PBS), or PBS alone (control). After 3 h, fish were infected with VHSV (TCID_50_ = 5.2 × 10^3^). Survival rates and VHSV G mRNA expression levels were monitored over 1 to 8 d postinfection as indicated. Fish injected with PBS only served as the negative control. **P < 0.01. (*E*) Blocking assay of VHSV entry by His-LjCD9 and His-LjCD9-EC2 proteins. VHSV was preincubated with His-LjCD9 (100 or 500 ng), His-LjCD9-EC2 (100 or 500 ng), or His protein (500 ng) at 4 °C for 4 h, then added to LJB cells for 4 h; VHSV G expression was detected by RT-qPCR. (*F* and *G*) Effect of LjCD9 protein on VHSV infection in vivo. Survival rates of sea perch intraperitoneally infected with premixtures of VHSV and His-LjCD9 or His protein as control for 4 h at 4 °C. PBS-injected group served as the negative control. The cumulative survival rate was determined from 1 to 9 d postinfection. (*H*) RT-qPCR analysis of VHSV *G* and *N* mRNA in LjCD9-overexpressing HEK293T cells infected with VHSV for 4 h at 28 °C. (*I*) Transmission electron microscopy of VHSV-infected HEK293T cells transfected with Flag-LjCD9 or empty vector. Black arrows indicate viral particles. Bar = 500 nm. (*J*) Immunofluorescence staining of VHSV-infected HEK293T cells expressing Flag-LjCD9. VHSV G (green) and LjCD9 (red) were detected with specific antibodies; nuclei stained with DAPI. PBS was treated as control. Images shown are representative of three independent experiments. Bar = 10 μm. Each biological replicate (n = 4) contains 3,000 analyzed cells. (*K*) Colocalization of DID-labeled VHSV virions (purple) with GFP-LjCD9 (green) in HEK293T cells after 4 h incubation (28 °C). Bar = 10 μm. (*L* and *M*) Effect of zebrafish CD9 knock out on VHSV infection in vivo. Survival rates and mRNA expression of wild-type (WT) or zCD9 knock out (zCD9^−/−^) zebrafish intraperitoneally infected with VHSV at 15 °C. PBS-injected fish as control. The cumulative survival rate was determined from 1 to 8 d postinfection. **P < 0.01. (*N*) VHSV *G* mRNA levels in WT and zCD9^−/−^ zebrafish cells at 4, 12, and 24 hpi. **P < 0.01. (*O*) VHSV *G* mRNA levels in zCD9^−/−^ zebrafish cells rescued with Flag-LjCD9 or empty vector, followed by VHSV infection for 24 h. **P < 0.01.

To confirm LjCD9 acts as a receptor for VHSV infection, we tested whether anti-CD9 antibody and LjCD9 proteins could block VHSV infection in vitro and in vivo. As shown in Fig. 2*A*, preincubation of LJB cells with anti-CD9 antibody markedly reduced VHSV entry into LJB cells, compared to control (IgG-treated cells), as demonstrated by the significant downregulation of VHSV *G*, *M*, *N*, and *P* expression at 2 and 4 hpi. In vivo, sea perch was injected with anti-CD9 antibody at 4 h prior to VHSV infection, which resulted in a significant reduction in VHSV-induced fish mortality (Fig. 2 *B* and *C*), VHSV *G* mRNA level (Fig. 2*D*) and aggregates of pyknotic nuclei, multifocally swollen or vacuolates in liver, brain, spleen, and intestine tissues (*SI Appendix*, Fig. S3*A*). Similarly, pretreatment of VHSV with purified His-LjCD9 or His-LjCD9-EC2 proteins inhibited VHSV *G* expression in LJB cells, respectively (Fig. 2*E*). Furthermore, pretreatment of VHSV with purified His-LjCD9 protein increased fish survival rate in a dose-dependent manner (Fig. 2 *F* and *G*).

Next, HEK293T cells [nonsusceptible but permissive to VHSV (34, 35)] were transfected with LjCD9 and then infected with VHSV. First, the presence of VHSV *N* and *G* mRNA was detected in LjCD9-overexpressing HEK293T cells using both RT–qPCR and RT–PCR (Fig. 2*H* and *SI Appendix*, Fig. S3*B*). In contrast, empty vector-transfected cells did not exhibit such mRNA expression. Subsequently, VHSV entry and replication in LjCD9-overexpressing HEK293T cells were confirmed via transmission electron micrograph observation, revealing a significant distribution of viral particles within the cytoplasm of LjCD9-overexpressing HEK293T cells (Fig. 2*I*). Immunofluorescence staining further showed that VHSV G protein colocalized with LjCD9 in VHSV-infected HEK293T cells at 24 hpi (Fig. 2*J*). Moreover, snapshots at 4 h post-VHSV infection revealed the colocalization of DID-VHSV particles and LjCD9 protein on the cell membrane, followed by internalization into the cell cytoplasm (Fig. 2*K* and *SI Appendix*, Fig. S4). These findings provide evidence that LjCD9 functions as an entry receptor for VHSV, playing a crucial role not only in attachment but also in internalization.

### CD9 Knockout Greatly Attenuates VHSV Infection In Vivo and In Vitro.

To elucidate the role of CD9 in VHSV infection in vivo, we compared viral replication and host survival rates between wild-type (WT) and CD9-knockout (zCD9^−/−^) zebrafish (*SI Appendix*, Fig. S5*A*). Groups of 30 WT and zCD9^−/−^ zebrafish were intraperitoneally injected with VHSV. zCD9^−/−^ zebrafish exhibited a drastically higher survival rate compared to WT counterparts (95% survival vs. 0%, *P* < 0.01) (Fig. 2*L*). Quantitative analysis showed a markedly reduced viral gene expression in VHSV-infected zCD9^−/−^ zebrafish compared to WT counterparts (Fig. 2*M*). Histopathological analysis revealed characteristic multifocal swelling and vacuolation in the intestines, liver, eyes, and brain (*SI Appendix*, Fig. S5*B*) of VHSV-infected WT zebrafish, which were notably absent in zCD9^−/−^ zebrafish. Consistent with these findings, in vitro experiments using cultured cells derived from WT and zCD9^−/−^ zebrafish demonstrated significantly reduced VHSV plaque formation in zCD9^−/−^ cells, as indicated by crystal violet staining (*SI Appendix*, Fig. S5*C*). RT-qPCR analysis further confirmed significant reduction of VHSV *G* and *M* mRNA in zCD9^−/−^ zebrafish cells at 4, 12, and 24 hpi (Fig. 2*N* and *SI Appendix*, Fig. S5*D*). Importantly, ectopic reexpression of zebrafish CD9 in zCD9^−/−^ cells rescued VHSV *G* and *M* gene expression in a dose-dependent manner (Fig. 2*O* and *SI Appendix*, Fig. S5*E*), thereby functionally restoring susceptibility to VHSV infection. These results demonstrate the critical role of fish CD9 in facilitating VHSV infection.

### CD9 Functions as a Common Receptor for Other Rhabdoviruses, Such as SCRV and VSV, across Genera.

**Fig. 3.**
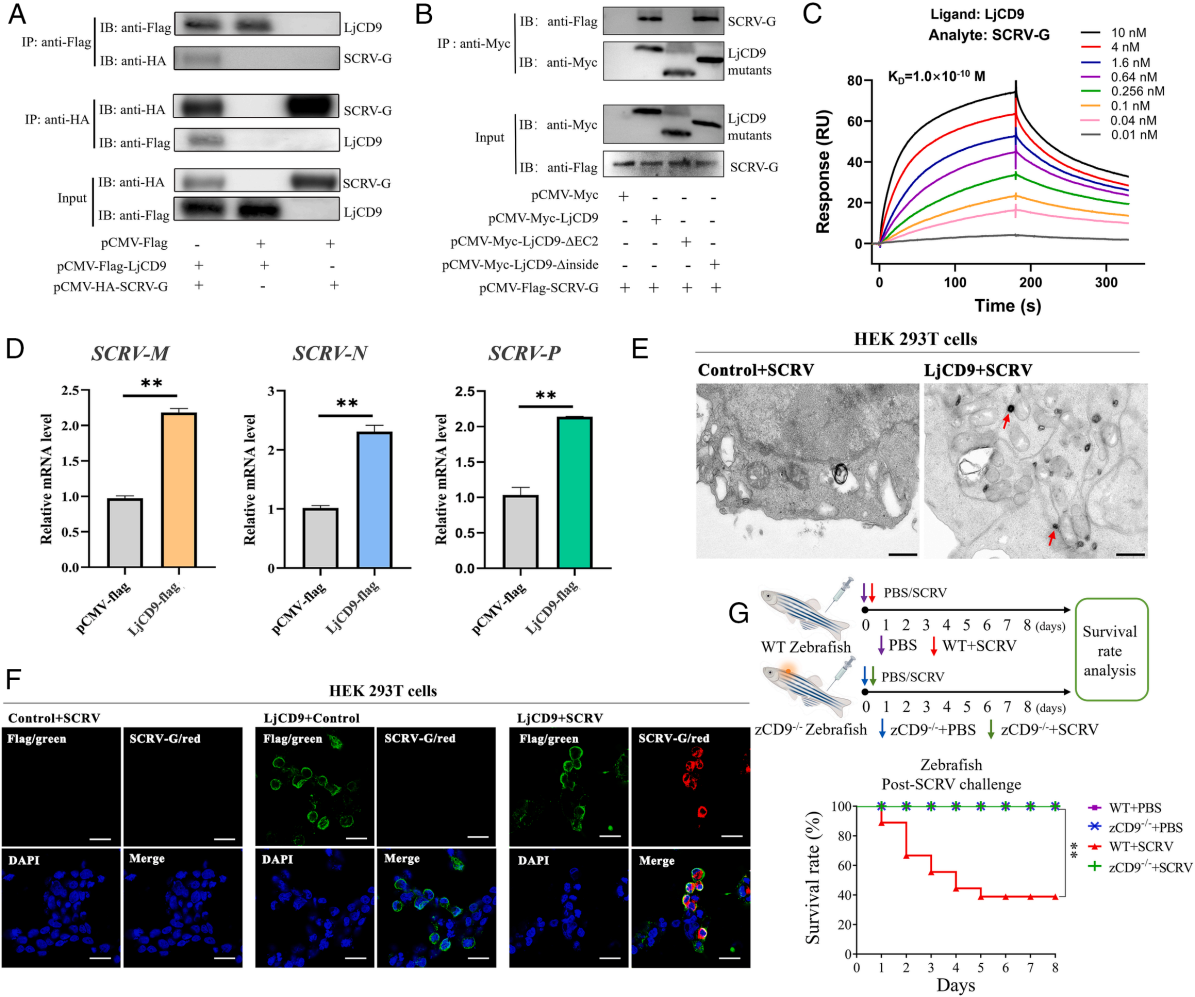
LjCD9 facilitates SCRV invasion. (*A*) Co-IP of LjCD9 and SCRV G. Cell lysates from HEK293T cells cotransfected with the LjCD9 and SCRV G plasmids were immunoprecipitated using the anti-Flag magnetic beads or anti-HA magnetic beads. (*B*) Co-IP of SCRV G with LjCD9 truncated mutants. A series of LjCD9 mutants and SCRV G were cotransfected into HEK293T cells for 24 h and subjected to immunoprecipitation with the anti-Myc magnetic beads, followed by immunoblotting analysis. (*C*) SPR analysis of SCRV G binding to immobilized LjCD9 protein. (*D*) SCRV replication in LjCD9-overexpressing HEK293T cells. Relative mRNA levels of SCRV *M*, *N*, and *P* measured by RT-qPCR at 4 hpi. Data = mean ± SEM; n = 3 biological replicates. (*E*) Transmission electron microscopy of SCRV-infected HEK293T cells transfected with empty vector or Flag-LjCD9 plasmid. SCRV virions (red arrow) are indicated. Bar = 500 nm. (*F*) Immunofluorescence analysis of SCRV infection in Flag-LjCD9-transfected HEK293T cells. LjCD9 (green; anti-Flag), SCRV G (red; anti-SCRV G), nuclei (blue; DAPI). Bar = 20 μm. Images represent three independent experiments; 3,000 cells/replicate analyzed (n = 4). (*G*) Survival rates of WT or CD9^−/−^ zebrafish intraperitoneally infected with SCRV at 28 °C as indicated. PBS-injected fish served as control. The cumulative survival rate was determined from 1 to 8 d postinfection. **P < 0.01.

To determine whether CD9 also serves as a common receptor for other rhabdoviruses across genera, we first compared the similarity between the G proteins of SCRV and VHSV (*SI Appendix*, Fig. S6), and then investigated the potential relationship between LjCD9 and SCRV G. As shown in Fig. 3 *A* and *B*, SCRV G could interact with LjCD9 protein by binding to the EC2 motif. SPR analysis revealed a dissociation constant (K_D_) of ~10^−10^ M (0.1 nM) for the LjCD9-SCRV G interaction (Fig. 3*C*), suggesting that LjCD9 may act as a receptor for SCRV. To validate this, we performed gain-of-function experiments in HEK293T cells, which appear nonsusceptible but permissive to SCRV (35, 36). Ectopic expression of LjCD9 rendered these cells susceptible to SCRV infection, as evidenced by the detection of SCRV *M*, *N*, and *P* gene transcripts (Fig. 3*D*). Similarly, transmission electron micrograph confirmed the presence of SCRV particles in infected LjCD9-overexpressing HEK293T cells, while control cells remained uninfected (Fig. 3*E*). Immunofluorescence further showed colocalization of SCRV G and LjCD9 proteins in SCRV-infected HEK293T cells at 24 hpi (Fig. 3*F*). Furthermore, zCD9^−/−^ zebrafish exhibited a significantly higher survival rate compared to WT counterparts after SCRV infection (100% survival vs. 40%, *P* < 0.01) (Fig. 3*G*). These results indicated that LjCD9 acts as a viral receptor for SCRV.

**Fig. 4.**
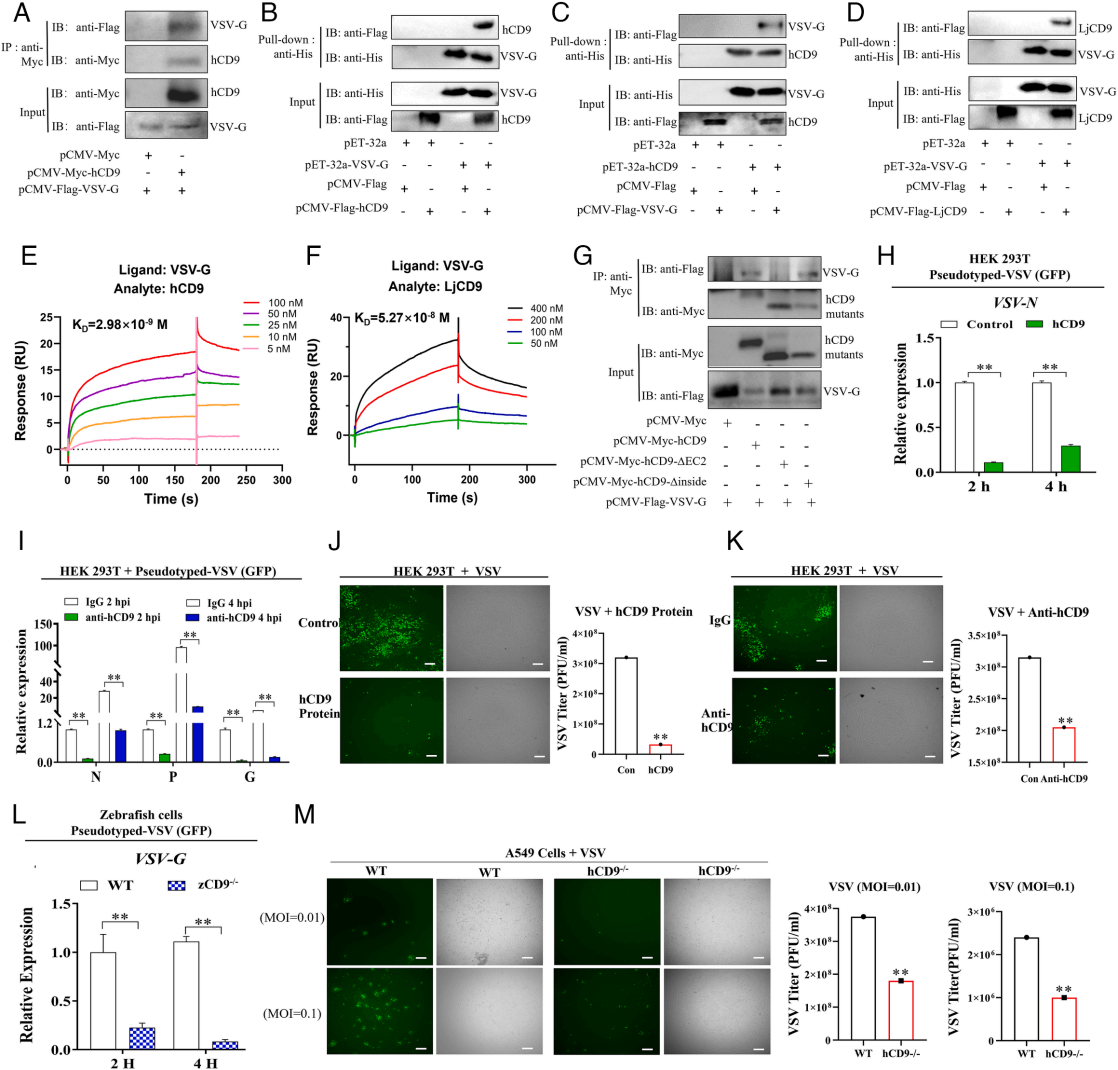
hCD9 facilitates VSV invasion. (*A*) Co-IP of hCD9 and VSV G. HEK293T cells were transfected with various plasmids as indicated. The cell lysates were then subjected to immunoprecipitation using anti-Myc magnetic beads. (*B* and *C*) hCD9 pulls down VSV G. The lysates of HEK293T cells transfected with indicated plasmids were pulled down with purified His-VSV-G protein (*B*), His-hCD9 (*C*) or His proteins using anti-His magnetic beads, and immunoblotted with anti-His and anti-Flag antibodies, respectively. (*D*) LjCD9 pulls down VSV G. The lysates of HEK293T cells transfected with indicated plasmids were pulled down with purified His-VSV-G or His proteins using anti-His magnetic beads, and immunoblotted with anti-His and anti-Flag antibodies, respectively. (*E* and *F*) SPR analysis of hCD9 or LjCD9 binding to immobilized VSV-G protein. VSV-G protein was immobilized on a CM5 sensor chip, and hCD9 (*E*) or LjCD9 (*F*) protein was passed at the indicated concentrations. (*G*) Co-IP of VSV G with hCD9 truncated mutants. A series of hCD9 mutants and VSV G were cotransfected into HEK293T cells for 24 h and subjected to immunoprecipitation with the anti-Myc magnetic beads, followed by immunoblotting analysis. (*H*) hCD9 protein blocks VSV entry. pseudotyped-VSV (GFP) was incubated with purified hCD9 (500 ng) or His (500 ng) proteins as control for 4 h at 4 °C. The virus-protein mix was then added to HEK293T cells for additional 4 h, followed by washing with PBS and detection of VSV N expression. (*I*) Anti-CD9 antibody inhibits VSV entry into HEK293T cells. HEK293T cells were incubated with commercial anti-human CD9 antibody (1:50) for 4 h and then infected with pseudotyped-VSV (GFP) for 2 or 4 h at 28 °C. Subsequently, the cells were washed with PBS and harvested for mRNA expression analysis of VSV *N*, *P,* and *G*. (*J*) Fluorescence and bright field images and virus titer assay of HEK293T cells infected with hCD9 and wild-type VSV mix for 12 h, Bar = 10 μm. (*K*) Fluorescence and bright field images and virus titer assay of HEK293T cells first incubated with commercial anti-human CD9 antibody or IgG as control, followed by wild-type VSV infection for 12 h. Bar = 10 μm. (*L*) The VSV *G* mRNA expression level in WT or CD9^−/−^ zebrafish cells infected with VHSV at 2 and 4 hpi. **P < 0.01. (*M*) Fluorescence and bright field imaging and virus titer assay of WT/hCD9^−/−^ A549 cells infected with wild-type VSV (MOI = 0.01 or 0.1) for 12 h. Data are representative of three independent experiments.

VSV, another rhabdovirus, is known to infect a broad range of organisms, including insects, fish, and mammals, and their cultured cells (37). To explore whether CD9 contributes to VSV tropism, we first confirmed the susceptibility of fish-derived cells (EPC, FHM, and LJB) to pseudotyped VSV (expressing GFP) via fluorescence detection at 24 hpi, indicating that VSV can infect fish (*SI Appendix*, Fig. S7*A*). We then examined the interaction between VSV G and CD9 proteins. Co-IP assays demonstrated that VSV G protein interacted with human CD9 (hCD9) (Fig. 4*A*). The interactions of VSV G and hCD9, as well as LjCD9 protein were confirmed by in vitro pull-down assays (Fig. 4 *B*–*D*). SPR analysis further validated the direct binding of VSV G to hCD9, revealing VSV G bound hCD9 with high affinity (K_D_ = 2.98 nM) (Fig. 4*E*) and LjCD9 with moderate affinity (K_D_ = 52.77 nM) (Fig. 4*F*). Domain mapping indicated that the EC2 motif of hCD9 is crucial for interaction with VSV G, while the intracellular region was dispensable (Fig. 4*G*).

To confirm CD9’s critical role as a VSV entry receptor, we performed a series of validation experiments. Preincubation of pseudotyped VSV (GFP) with purified hCD9 protein significantly inhibited infection in HEK293T cells (Fig. 4*H*). Similarly, antibody blocking with anti-CD9 antibody reduced pseudotyped VSV (GFP) entry into HEK293T cells at 2 and 4 hpi compared to control IgG (Fig. 4*I*). Moreover, the wild-type VSV (MOI = 0.1) infection and viral titers were also inhibited following incubation with either hCD9 protein (Fig. 4*J*) or anti-CD9 antibody (Fig. 4*K*) in HEK293T cells. Given the susceptibility of zebrafish to VSV (38), we compared the VSV infection in zCD9^−/−^ and WT zebrafish cells. zCD9^−/−^ zebrafish cells displayed notably decreased pseudotyped VSV (GFP) G expression (Fig. 4*L*), cytopathic effects (CPE), and plaque formation (*SI Appendix*, Fig. S7*B*). Further evidence came from hCD9-knockout (hCD9^−/−^) A549 cells, where hCD9 deletion significantly reduced VSV infection and markedly suppressed viral titers (Fig. 4*M*), further supporting CD9’s conserved role in VSV entry across species.

### LjCD9 Facilitates VHSV Entry via the CME and CavME Pathways.

**Fig. 5.**
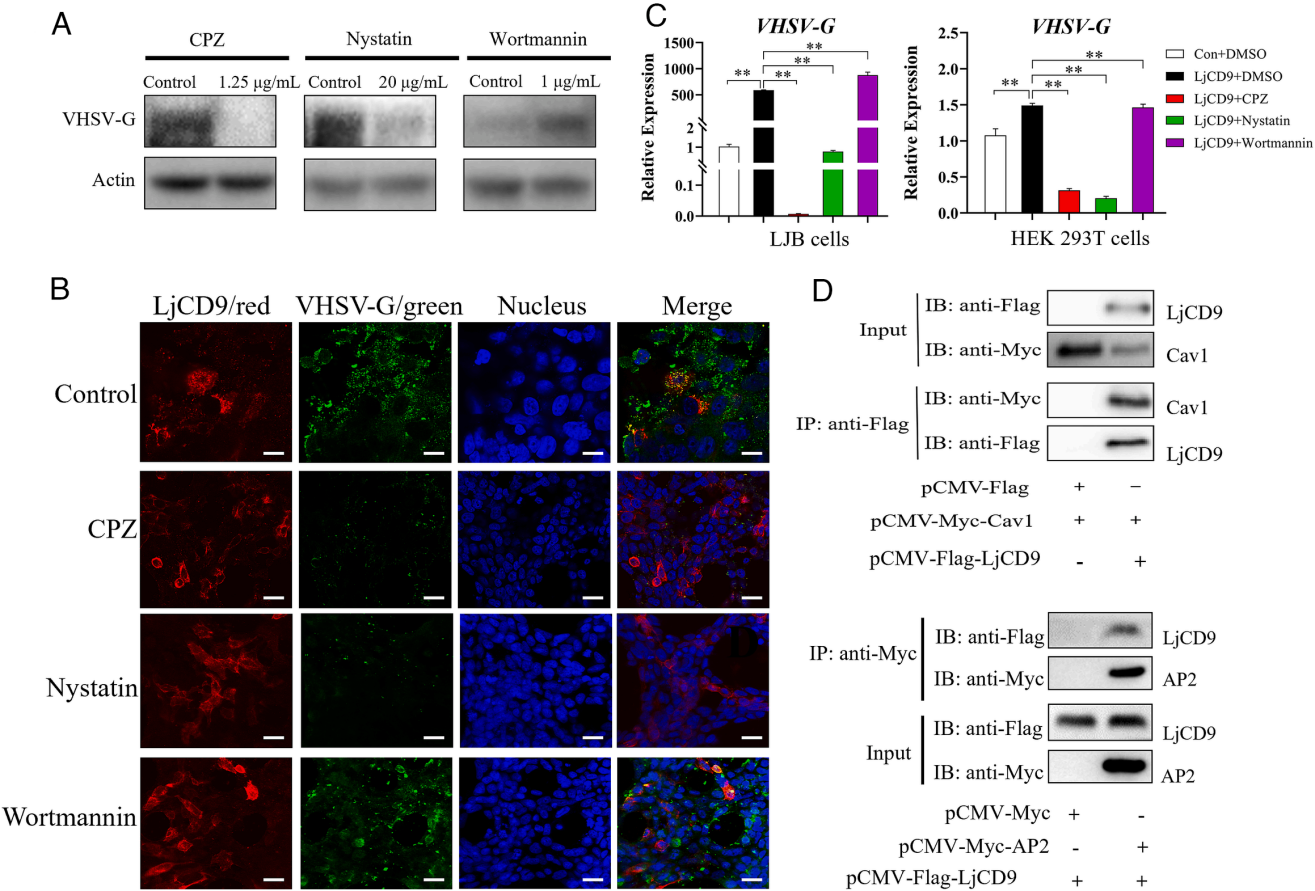
VHSV entry into LJB cells through clathrin-mediated and caveolae-mediated endocytosis. (*A*) Inhibition of VHSV entry by endocytic pathway blockers. LJB cells pretreated with clathrin inhibitors (chlorpromazine [CPZ]), caveolae inhibitors (Nystatin), or micropinocytosis inhibitor (wortmannin) were infected with VHSV (4 h, inhibitors present), washed, and the VHSV G protein was further detected by immunoblotting following 20 h incubation. **P < 0.01; Bar = 10 μm. (*B*) LjCD9 overexpression enhances endocytosis-dependent VHSV infection. Immunofluorescence analysis of VHSV entry in inhibitors-treated HEK293T cells. Flag-LjCD9-transfected cells pretreated with inhibitors were infected with VHSV (4 h), washed, incubated 20 h, and stained for VHSV G (green; anti-VHSV G) and LjCD9 (red; anti-Flag). Bar = 10 μm. (*C*) LjCD9-transfected LJB and HEK293T cells pretreated with indicated inhibitors were infected with VHSV (4 h, inhibitors present), washed, incubated 20 h, and analyzed for VHSV *G* mRNA by RT-qPCR. **P < 0.01. (*D*) Co-IP of LjCD9 with caveolin-1 (Cav1) and AP2. HEK293T cells cotransfected with Flag-LjCD9 and Myc-Cav1/AP2 plasmids were immunoprecipitated with anti-Flag or anti-Myc beads, followed by Western blot analysis.

To investigate the mechanism by which LjCD9 mediates VHSV entry, we first characterized the endocytic pathways utilized by VHSV in LJB cells. Treatment with clathrin-mediated endocytosis (CME) inhibitors (CPZ, Dynasore) and caveolin-mediated endocytosis (CavME) inhibitors (MβCD, Nystatin) (*SI Appendix*, Fig. S8*A*) dose-dependently decreased VHSV *G* mRNA levels in LJB cells, by contrast, the micropinocytosis inhibitor wortmannin exerted no significant effect (*SI Appendix*, Fig. S8*B*). Consistently, VHSV G protein expression was significantly reduced following pretreatment with CPZ and Nystatin (Fig. 5*A*), or Dynasore and MβCD (*SI Appendix*, Fig. S8*C*). Additionally, Fluorescence imaging further revealed diminished VHSV G signals in cells treated with clathrin or caveolin-1 inhibitors, compared to untreated controls (*SI Appendix*, Fig. S8*D*). These results indicate that VHSV entry into LJB cells relies primarily on the CME and CavME pathways, with no apparent involvement of micropinocytosis.

Next, we explored whether LjCD9 facilitates VHSV entry through these identified pathways. We subjected LjCD9-overexpressing LJB and HEK293T cells to endocytic inhibitors for 4 h prior to VHSV infection. CME and CavME inhibitors significantly attenuated LjCD9-facilitated viral entry in both cell lines, consistent with reduced VHSV G fluorescence signals and mRNA expression in inhibitors-treated, LjCD9-overexpressing HEK293T cells (Fig. 5 *B* and *C* and *SI Appendix*, Fig. S8*E*). Furthermore, Co-IP and IF demonstrated the coprecipitation and colocalization of LjCD9 with caveolin-1 and AP2 (a CME marker) (Fig. 5*D* and *SI Appendix*, Fig. S8*F*). These findings indicated that LjCD9 plays a crucial role in facilitating the entry of VHSV into cells via the CME and CavME pathways.

### NTZ Exhibits Anti-VHSV Activity by Competitively Binding to VHSV G.

**Fig. 6.**
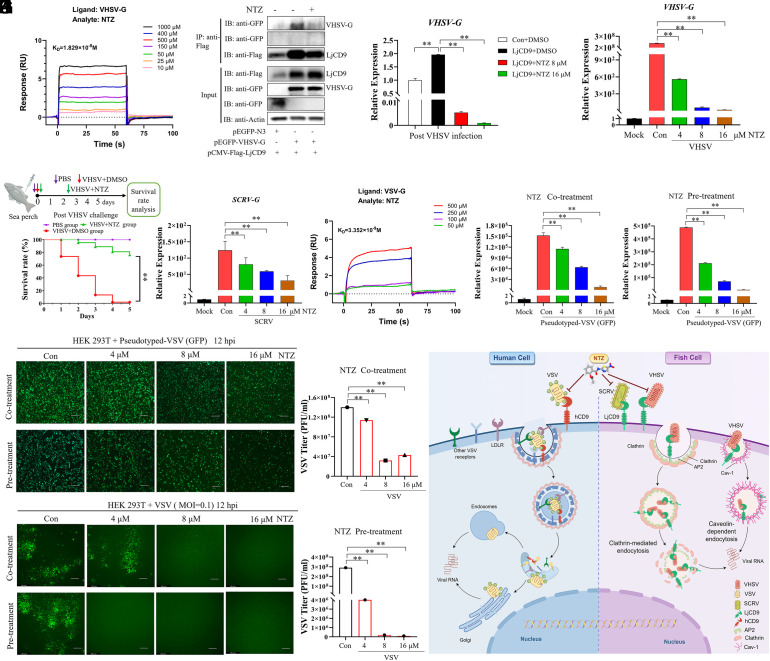
NTZ inhibits VHSV, SCRV, and VSV infection. (*A*) SPR analysis of direct binding between VHSV G protein and NTZ. (*B*) NTZ likely interferes with VHSV G-LjCD9 interaction. HEK293T cells transfected with Flag empty vector or Flag-LjCD9 plasmids and VHSV G were treated with NTZ or DMSO as a control, and subjected to immunoprecipitation with anti-Flag antibody. (*C*) NTZ inhibited LjCD9-facilitated VHSV infection. LjCD9-overexpressed LJB cells were infected with VHSV and treated with NTZ (8 and 16 μM) for 4 h, and then analyzed for VHSV *G* mRNA expression using RT-qPCR. (*D*) NTZ inhibits VHSV infection in a dose-dependent manner. LJB cells were infected with VHSV and treated with different concentrations of NTZ for 36 h, followed by RT-qPCR. DMSO (Con) were used as control. **P < 0.01. (*E*) Survival rate of VHSV-infected sea perch with NTZ treatment as indicated. Fish was intraperitoneal coinjected with VHSV and NTZ or DMSO, respectively (n = 30). PBS-injected group as negative control. **P < 0.01. (*F*) NTZ inhibits SCRV infection in a dose-dependent manner. LJB cells were infected with SCRV and treated with different concentrations of NTZ for 36 h, and analyzed using RT-qPCR for SCRV *G* gene expression. (*G*) SPR analysis of direct binding between VSV G and NTZ. VSV G protein was coupled to a CM5 chip, and NTZ was passed at the indicated concentrations. Data are representative of three independent experiments. (*H*–*K*) Inhibitory effects of NTZ on VSV infection. HEK293T cells were infected by pseudotyped-VSV (GFP) (*H* and *I*), or wild-type VSV (*J* and *K*), with cotreating with dose-dependent NTZ or preincubating with dose-dependent NTZ for 3 h, followed by RT-qPCR or virus titer assay at 12 hpi. **P < 0.01. Bar = 200 μm. (*L*) Schematic model of how CD9 facilitates the entry of VHSV, SCRV, and VSV into host cells, and facilitates VHSV internalization by clathrin-and caveolae-mediated endocytosis. CD9 facilitates VHSV, SCRV, and VSV entry by binding their G proteins, and triggering clathrin- or caveolae-mediated endocytosis to mediate VHSV infection. While, likely by interfering CD9-G interaction, NTZ inhibits the infection of VHSV, SCRV, and VSV. The graphic is created by figdraw.com.

Given that LjCD9 functions as an entry receptor by binding to the domain IV of G, we employed the G domain IV structure as a reference to screen for small molecules that might interfere with this interaction. Molecular docking analyses predicted NTZ might bind to domain IV of VHSV G, SCRV G, and VSV G, overlapping with the LjCD9 binding sites on G protein (*SI Appendix*, Fig. S9*A*). This suggested NTZ might interferes with VHSV G-LjCD9 interaction during viral entry. To test this hypothesis, we conducted two key experiments. First, SPR analysis validated the direct binding of NTZ to VHSV G protein with high affinity (K_D_ = 18.29 nM; 10^−8^ M) (Fig. 6*A*), supporting the idea that NTZ likely interferes with the VHSV G-LjCD9 protein interaction. Next, HEK293T cells were cotransfected with VHSV G and LjCD9 expression plasmids and treated with either NTZ or DMSO (control). Co-IP assays showed NTZ significantly reduced the amount of LjCD9 coimmunoprecipitated with VHSV G (Fig. 6*B*). Concurrently, in LjCD9-overexpressing LJB cells infected with VHSV, NTZ treatment led to a marked decrease in VHSV *G* mRNA levels (Fig. 6*C*). We then evaluated the direct inhibitory effect of NTZ on VHSV infection. NTZ exhibited minimal cytotoxicity (CC_50_ = 58.98 ± 4.71 μM) with potent inhibitory effects against VHSV infection (EC_50_ = 3.52 ± 0.58 μM) (*SI Appendix*, Fig. S9 *B*–*D*). Consistent with this, NTZ also significantly and dose-dependently suppressed VHSV *G* mRNA expression (Fig. 6*D*), crystal violet staining (*SI Appendix*, Fig. S9*E*), and mitigated virus-induced CPEs (*SI Appendix*, Fig. S9*F*). Further mechanistic assays confirmed that NTZ strongly inhibited VHSV binding, internalization, and entry into LJB cells compared to DMSO controls (*SI Appendix*, Fig. S9*G*). In vivo, NTZ significantly improved sea perch survival rates following VHSV challenge (76.2% vs. 1.88%, *P* < 0.01) (Fig. 6*E*). Collectively, these data demonstrate NTZ might block VHSV entry by targeting G domain IV, offering repurposing potential as an antiviral agent against VHSV infection.

### NTZ Exhibits Potent Antiviral Activity against VSV and SCRV.

Given NTZ’s demonstrated ability to block VHSV entry by targeting the conserved domain IV of the viral G protein, we investigated its efficacy against other rhabdoviruses, specifically SCRV and VSV. For SCRV, NTZ treatment of infected LJB cells resulted in a dose-dependent reduction in viral *G* mRNA expression (Fig. 6*F*). Against VSV, SPR analysis confirmed direct binding of NTZ to VSV G protein with high affinity (K_D_ = 3.35 nM) (Fig. 6*G*). In HEK293T cells, NTZ exhibited minimal cytotoxicity (CC_50_ = 28.9 ± 3.8 μM) (*SI Appendix*, Fig. S9*H*). NTZ treatment, either coadministered with pseudotyped VSV (GFP) (cotreatment) or preincubated with the virus (pretreatment), significantly reduced viral infection and *G* mRNA expression in HEK293T cells in a dose-dependent manner (Fig. 6 *H* and *I* and *SI Appendix*, Fig. S10*A*). Consistent results were observed in LJB cells, where NTZ inhibited pseudotyped VSV (GFP) infection (*SI Appendix*, Fig. S10*B*). Furthermore, in HEK293T cells infected with VSV (MOI = 0.1), NTZ treatment reduced particle formation and viral titers (Fig. 6 *J* and *K* and *SI Appendix*, Fig. S10*C*). These findings confirm NTZ’s broad-spectrum antiviral efficacy against rhabdoviruses beyond VHSV, including VSV and SCRV, likely through competitive inhibition of G protein–receptor interactions.

## Discussion

Despite the broad host range and significant impacts of rhabdoviruses, particularly on global aquaculture and veterinary health, the cellular receptors mediating their entry remain poorly defined. In this study, we uncover the tetraspanin CD9 as a conserved common receptor for rhabdoviruses across genera, including *Novirhabdovirus* (VHSV), *Vesiculovirus* (VSV), and *Siniperhavirus* (SCRV). Our study provides critical insights into a shared entry mechanism among rhabdoviruses and highlights CD9 as a promising target for broad-spectrum antiviral interventions.

Previous studies have identified rhabdoviruses receptors, such as LDLR for VSV in mammals and neural cell adhesion molecule (39), nicotinic acetylcholine receptor (nAChR) (40), and p75NTR (41) for RBAV, but these fail to explain the broad host range of rhabdoviruses. Our findings identify LjCD9 (fish CD9) as the first validated entry receptor for VHSV. Furthermore, we confirmed its cross-genus activity—spanning *Novirhabdovirus* and *Siniperhavirus,* demonstrating that CD9’s receptor function is not restricted to a single viral clade. The conservation of CD9-G interactions across distantly related fish rhabdoviruses (VHSV, IHNV, and SCRV) suggests an evolutionary adaptation to exploit a ubiquitous host membrane protein in aquatic hosts. In contrast, our results show that VHSV G does not interact with hCD9 (*SI Appendix*, Fig. S11), providing a possible explanation for the restricted infectivity of fish rhabdoviruses in mammalian hosts. Building on findings in fish, further studies reveal hCD9 functions as a novel receptor for VSV in mammals. VSV’s ability to engage LDLR and CD9 might provide a mechanistic explanation for its cross-species infectivity, from insects (CD9 homologs had been identified in multiple insect species) to mammals, highlighting evolutionary plasticity in virus–host interactions. Tetraspanins are ancient molecules, with CD9-like proteins identified in teleost fish and mammals, making them ideal targets for viral adaptation (42–44). The ability of VSV to bind CD9 from distantly related species (e.g., sea perch and human) supports a “receptor-sharing” model that facilitates cross-species transmission (45), a critical consideration for emerging viruses in aquaculture and zoonotic contexts. Overall, the utilization of CD9 as a receptor across diverse rhabdovirus species likely reflects a convergent evolutionary strategy for rhabdoviruses to exploit conserved membrane proteins across hosts.

The glycoprotein G of rhabdoviruses, composed of four domains (I-IV), mediates receptor binding and membrane fusion (16, 46). Our domain mapping studies show that the interaction between CD9 and rhabdovirus G is mediated by the EC2 motif of CD9 and the domain IV of G. This contrasts with RABV G, which employs domains I and III of G to bind p75NTR and nAChR (47–49), respectively, illustrating functional divergence in receptor engagement among rhabdoviruses. SPR analysis reveals high-affinity interactions between LjCD9 and VHSV G (K_D_ = 0.12 nM) and SCRV G (K_D_ = 0.10 nM), while LjCD9-VSV G interaction is less avid (K_D_ = 52.77 nM). This variation in binding affinity may reflect the evolutionary adaptation of viruses to utilize CD9 as a primary or secondary receptor, depending on host availability.

A typical tetraspanin consists of four transmembrane domains, a small extracellular loop, and a large extracellular loop (EC2) (50). The EC2, comprising a conserved region with 2 to 6 invariant cysteine residues and a variable region, plays a critical role in protein–protein interactions, including viral proteins (51–53). Our findings demonstrate that the G proteins of VHSV, VSV, and SCRV all interact with the EC2 of CD9 (e.g., LjCD9 and hCD9). This conserved interaction across distinct rhabdoviruses highlights the evolutionary preservation of EC2 as a binding hub for viral entry. Notably, while VSV can infect both mammals and fish, fish-specific rhabdoviruses (such as VHSV and SCRV) exhibit restricted host ranges, a disparity likely rooted in sequence variations within the EC2. These variations may modulate viral tropism by influencing the specificity of G protein–EC2 interactions. The conserved utilization of the EC2 in fish and mammalian CD9 for mediating rhabdovirus binding suggests that targeting this pathway could yield broad-spectrum inhibitors effective against both current and emerging rhabdoviruses.

Leveraging the conserved CD9-viral G protein interactions, we identified NTZ, an FDA-approved antiparasitic drug for protozoan-caused diarrhea, as a broad-spectrum competitive inhibitor of rhabdovirus entry. Notably, NTZ exhibits cross-genus efficacy against phylogenetically distinct rhabdovirus genera, including *Novirhabdovirus*, *Siniperhavirus*, and *Vesiculovirus*, supporting its potential as a broad-spectrum, pan-rhabdoviral antiviral agent. Importantly, NTZ’s efficacy against other diverse viruses, including MERS-CoV and porcine reproductive and respiratory syndrome virus (54, 55), underscores its utility as a repurposed antiviral agent. Our data suggest NTZ might act by interfering with the CD9-G protein interaction, a key step in viral entry. However, definitive elucidation of the molecular mechanism by which NTZ inhibits the G-CD9 interaction would require additional evidence, such as cocrystal structures of the NTZ-G protein complex, mutagenesis of predicted binding residues, or other direct structural analyses. While NTZ’s in vivo toxicity and pharmacokinetics require further validation in larger animal models, its dual activity against fish and mammalian pathogens highlights NTZ’s significant potential as a broad-spectrum countermeasure against rhabdoviruses.

CD9’s primary receptor role is firmly validated for three rhabdovirus genera, however, its role in other genera (e.g., *Lyssavirus* and *Perhabdovirus*) remains untested. Verifying whether CD9-mediated entry is a shared mechanism across the entire Rhabdoviridae family requires systematic investigation. Additionally, the potential crosstalk between CD9 and other coreceptors during rhabdovirus entry warrants further exploration to fully understand the complexity of rhabdovirus tropism. While our study identifies the CD9-EC2 domain and rhabdovirus G protein domain IV as key interacting regions, the precise molecular architecture of their interaction remains unresolved. Determining the high-resolution structure of the CD9-G protein complex, therefore, represents the most promising avenue forward. Cryoelectron microscopy (cryo-EM), which has successfully elucidated tetraspanin complexes (56) and critical viral entry mechanisms, including Rabies virus (48) and ACE2-SARS-CoV-2 spike (57), represents the optimal approach. Complementary methods, including X-ray crystallography and hydrogen–deuterium exchange mass spectrometry, would provide additional mechanistic insights (58). Structural data would enable rational design of broad-spectrum antivirals (small molecules, peptides, or antibodies) targeting the CD9-G interface and thus represent a critical frontier for understanding this host–pathogen interaction and developing next-generation antiviral strategies.

In summary (Fig. 6*L*), we identify tetraspanin CD9 as a conserved functional entry receptor for rhabdoviruses across multiple genera. Furthermore, we demonstrate that NTZ currently being repurposed and evaluated as an antiviral candidate of rhabdoviruses, likely interferes with the G-CD9 interaction. Our findings significantly enhance the understanding of rhabdovirus entry mechanisms and open avenues for developing broad-spectrum antiviral therapies.

## Materials and Methods

Detailed materials and methods are described in *SI Appendix*, SI Materials and Methods**. Plasmid construction, RNA extraction, RT-PCR and RT–qPCR, gene overexpression and knockdown, immunoblotting, Co-IP and pull-down, protein expression and purification, SPR analysis, immunofluorescence assays, viral infection, titration, labeling and detection, blocking assays, crystal violet staining, hematoxylin and eosin (HE) staining, cell viability assay, inhibitor assays, transmission electron microscopy, molecular AutoDocking, virus challenge, generation of CD9-deficient zebrafish, and generation of hCD9-knockout A549 cells by CRISPR/Cas9 gene editing were performed. Sequence alignment of the CD9 knockout construct used in this study is listed in *SI Appendix*, Table S1. Primer sequences of the genes used in this study are listed in *SI Appendix*, Table S2. GenBank accession numbers of genes and sequence information of mutants used in this study are listed in *SI Appendix*, Table S3.

## Supplementary Material

Appendix 01 (PDF)

## Data Availability

Study data are included in the article and/or *SI Appendix*.
