# Supplementary material for: Conserved use of tetraspanin CD9 as an entry receptor by rhabdoviruses spanning multiple genera
Source: Proc Natl Acad Sci U S A. 2026 Mar 11;123(11):e2530369123. doi: 10.1073/pnas.2530369123 (PMC12993960; doi:10.1073/pnas.2530369123)
Supplement: Supplementary file 1 — Appendix 01 (PDF) [file pnas.2530369123.sapp.pdf]

Supporting Information for

Conserved use of tetraspanin CD9 as an entry receptor by rhabdoviruses spanning multiple genera

Wanwan Zhang[1], Bingbing Sun[1], Hao Huang, Junyu Chen, Ying'an Liang, Wenxi Li, Qinyu Peng, Ping Zhang, Zhipeng Zhan, Xiaogang Yang, Lan Yao, Huiquan Chen, Eran Bacharach, Haifeng Li, Changjun Guo*, Meisheng Yi*, Kuntong Jia*

[1] Equally contributing author: Wanwan Zhang and Bingbing Sun.

* Corresponding authors: Changjun Guo, Meisheng Yi and Kuntong Jia.

Email: gchangj@mail.sysu.edu.cn (Changjun Guo), yimsh@mail.sysu.edu.cn (Meisheng Yi), jiakt3@mail.sysu.edu.cn (Kuntong Jia)

This PDF file includes:

Supplementary Materials and Methods
Supplementary Figures S1 to S12
Legends for Supplementary Figures S1 to S12
Supplementary Table S1 to S3

Supplementary Materials and Methods

Cells

Fish cell lines, including *L. japonicus* brain cell (LJB) (1), Epithelioma papulosum cyprini cell (EPC) (2), Fathead minnow cell (FHM) (3), and Mandarin fish fry (MFF-1) (4) cell lines were cultured in Dulbecco's modified Eagle's medium (DMEM, Gibco,12100061) supplemented with 15% fetal bovine serum (FBS, Gibco, A5256701), 100 U/mL penicillin and streptomycin (Gibco, 15140-122) in a humidified incubator at 28°C.

Human lung carcinoma epithelial cells (A549, ATCC CCL-185) (5) and human embryonic kidney cells (HEK293T, ATCC CRL-3216) (6) were cultured in DMEM supplemented with 10% FBS at 37°C with 5% CO_2. African green monkey kidney cells (Vero, ATCC CCL-81) (7) were maintained in DMEM supplemented with 5% FBS. All media were supplemented with 100 U/mL of penicillin and streptomycin.

Primary zebrafish cells were derived from WT or CD9-knockout zebrafish larvae (20 dpf). Larvaes were surface-sterilized in 75% ethanol, minced into small pieces dissection under aseptic conditions in phosphate-buffered saline (PBS), and dissociated in 0.25% trypsin-EDTA (Macklin, T6325) at 28°C for 15 min. The cell suspension was filtered through a 40-μm mesh, centrifuged (300 × g, 5 min), and resuspended in DMEM supplemented with 20% FBS and 4.76 g/L HEPES (Gibco,15630130) (pH 7.4), then incubated at gelatin-coated plates and maintained at 28°C in a humidified incubator. The culture medium was refreshed every 2–3 days until cells reached 90% confluence. Prior to experimental use, genotyping was performed to confirm the disruption of the zCD9 gene. zCD9 gene region was amplified by PCR using gRNA-specific primers (gRNA1-Forward primer (5'-3'): gatcccaagaccaagagttt; gRNA1-Reverse primer (5'-3'): ggggtctaataattcaggagag), and the PCR products were subsequently sequenced, and subjected to sequence alignments (Table S1).

Virus

VHSV (GenBank: MK598848.1) was isolated from diseased fish (8), and propagated in FHM cells, with titers determined by virus titration assay.

SCRV strain NH-2103 (GenBank: PQ066876.1) were isolated and stored in our laboratory as previously described (9). SCRV was propagated in MFF-1 cell line that was constructed and maintained in the laboratory as described (4).

Recombinant VSV expressing GFP (vesicular stomatitis virus [VSV]-GFP) (Indiana serotype, Mudd-Summers strain) was a gift from Professor Dongyan Jin (University of Hong Kong) (10). All viruses are kept in the -80°C freezer.

The recombinant VSV pseudotypes (>10^7 PFU/mL) (Cat. No. VB010000-9315gcp) were purchased from Yunzhou Biotechnology Co., Ltd. These pseudoviruses were constructed on a reverse

genetics platform in which the native VSV G gene was replaced with an enhanced green fluorescent protein (EGFP) reporter gene. Viral infectivity was restored through trans-complementation of the VSV G glycoprotein during production, resulting in particles displaying VSV G protein and carrying the EGFP gene (11). EGFP expression after infection serves as a direct readout of viral entry.

Regents

Primary antibodies, including anti-Flag (M20008L), anti-Myc (M20002L), anti-His (M20001L), anti-hemagglutinin (HA) (M20003L), and anti-actin (P30002L), were obtained from Abmart (Cambridge Science Park, UK). Antibodies against CD9 (ab236630) were obtained from Abcam (Beijing, China) and validated for cross-reactivity with fish CD9 (*SI Appendix*, Fig. S12). Horseradish peroxidase (HRP)-conjugated goat anti-mouse IgG (31430), HRP-conjugated goat anti-rabbit IgG (31460), Alexa Fluor 555-conjugated donkey anti-mouse IgG (H+L) (A-31570) and Alexa Fluor 488-conjugated goat anti-rabbit IgG (H+L) (A-21206) were obtained from Invitrogen (Carlsbad, CA, USA). All antibodies were diluted according to the manufacturer's instructions. Magnetic beads against His (HY-K0209), c-Myc (HY-K0206), and Flag (HY-K0207) were purchased from MedChemExpress (Monmouth Junction, NJ, USA). Isopropyl-1-thio-b-D-galactopyranoside (IPTG) (I6758) was obtained from Sigma–Aldrich (St. Louis, MO).

Chemical inhibitors were obtained from MedChemExpress, including CPZ (Cat. No. HY-12708), Dynasore (Cat. No. HY-15304), MβCD (Cat. No. HY-101461), Nystatin (Cat. No. HY-17409), and Wortmannin (Cat. No. HY-10197) and NTZ (Cat. No. HY-B0217).

Plasmid construction

All construct primer information referenced have been provided in Table S2. All genes GenBank Accession Number and mutants' sequences information have been provided in Table S3. The full-length sequences of *L. japonicus* CD9 (LjCD9), cavolin-1 (cav1), and AP2 were amplified by PCR and subsequently cloned and inserted into the pCMV-Flag/Myc vector (Clontech). LjCD9 deletion mutants (LjCD9Δinside, LjCD9ΔEC2 and LjCD9-EC2) were constructed by PCR and cloned into pCMV-Flag vector. CD9 from largemouth perch (MsCD9) was cloned and inserted into the pCMV-Flag vector. hCD9 and its deletion mutants (hCD9Δinside and hCD9ΔEC2) were constructed by PCR and cloned into pCMV-Myc vector.

VHSV G with GFP tags, VHSV G protein deletion mutants (Δdomain I–IV) and VHSV G-IV were constructed by PCR and cloned into pCMV-Flag vector. The SCRV G protein with Myc tags was obtained as described previously (12). The sequences of VSV G and IHNV G were synthesized by Tsingke Biotechnology Co., Ltd and inserted into the pCMV-Flag, pEGFP-N3 or pCMV-Myc vector.

RNA extraction, RT-PCR and RT–qPCR

Total RNA was extracted using TRIzol Reagent (Invitrogen, CA, USA) following the manufacturer's protocol. The RNA quantity was assessed using a NanoDrop spectrophotometer and agarose gel electrophoresis. Subsequently, the RNA was reverse transcribed into cDNA through the PrimeScript RT reagent kit with gDNA Eraser (TaKaRa).

RT-PCR was conducted by using cDNA as template for PCR amplification, and using gene-specific primers (Table S1) and DNA polymerase (Vazyme, P520-01) through 35 thermal cycles of denaturation, annealing, and extension. Products were detected by agarose gel electrophoresis.

RT-qPCR was conducted using cDNA as the template, which was mixed with Fast SYBR Green Master Mix (GHI Company, Cat No. 5678) on a LightCycler 480 II (Roche Applied Science, Germany). Each reaction had a total volume of 20 μL, containing 10 μL of SYBR Green Master Mix (Yeasen,11203ES03), 1 μL of gene-specific primers (Table S1), 2 μL of cDNA template, and 6 μL of nuclease-free water. The primer sequences were designed to span exon–exon junctions to avoid genomic DNA amplification. The RT-qPCR cycling conditions included initial denaturation at 95°C for 10 min, followed by 40 cycles of 95°C for 15 s and 60°C for 1 min. Melting curve analysis was performed to confirm the specificity of the PCR products. The relative expression levels of the target genes were calculated using the $2^{-\Delta\Delta Ct}$ method and normalized to the expression of a housekeeping gene (*β-actin*). Each sample was run in triplicate, and the data are presented as the mean ± SEM.

Gene overexpression and knockdown

Gene expression in cells was modulated by cytoplasmic plasmid transfection or siRNA knockdown. For plasmid transfection, the target cells were plated to achieve 60-80% confluence. The plasmid expressing the target gene was diluted in Opti-MEM (Gibco, 31985070) and mixed with Lipofectamine 8000 (Beyotime, C0533). After a 15-min incubation, the complexes were added to the cells for an incubation of 4-6 h. The transfection medium was then replaced with fresh growth medium, and gene expression was analyzed post transfection.

For siRNA knockdown, three small interfering RNAs (siRNAs) targeting LjCD9, namely, siRNA-1 (5'-CACCGATACCTGCAGGGA-3'), siRNA-2 (5'-GAGGAAGAGGAGGACCC-3'), siRNA-3 (5'-AAGAACAGGAAATCATT-3'), and control siRNA (NC) (5'-UUCUCCGAACGUGUCACGUTT-3'), were synthesized by RiboBio (Guangzhou, China). Cells were transfected with different siRNAs (50 or 100 nM) as described above, and the siRNA with the highest knockdown efficiency was chosen for subsequent experiment.

Immunoblotting, Co-IP and Pull-down

Total protein was extracted from cultured cells using lysis buffer (Beyotime, P0013) supplemented with protease and phosphatase inhibitors (MedChemExpress, HY-K0010). Protein concentration was quantified using a BCA Protein Assay Kit (Thermo Fisher Scientific, A65453) according to the manufacturer's instructions. Equal amounts of protein were separated by 10% SDS–PAGE and

electrotransferred onto polyvinylidene difluoride (PVDF) membranes (Millipore, 03010040001). The membranes were blocked with 5% nonfat dry milk in TBST (10 mM Tris-HCl, pH 7.5, 150 mM NaCl, 0.1% Tween-20) for 1 h at room temperature, then probed with primary antibodies overnight at 4°C. The primary antibodies included: anti-Flag (1:4,000), anti-β-actin (1:2,000), anti-Myc (1:4,000), anti-GFP (1:4,000), anti-His (1:4,000), anti-VHSV G (1:1,000), and anti-CD9 (1:1,000). After washing with TBST, membranes were incubated with HRP-conjugated secondary antibodies (1:10,000) for 1 h at room temperature. Protein bands were visualized using an enhanced chemiluminescence detection system (Millipore, USA) and imaged with a chemiluminescence imager (Sage Creation, China).

For Co-IP experiments, cell lysates were incubated with anti-Flag/Myc magnetic beads at 4°C overnight under gentle rotation. The beads were washed five times with ice-cold lysis buffer to remove unbound proteins, and immune complexes were eluted with 2× SDS loading buffer at 95°C for 10 min. Eluted proteins were subjected to Western blot analysis as described above to detect interacting proteins.

The pull-down assay was performed as previously described (13). The His-tag magnetic beads were combined with His-fused protein and incubated at room temperature for 4 h. Afterwards, the beads were exposed to protein lysates from plasmid-transfected HEK293T cells and incubated overnight at 4°C. The samples were subsequently analyzed using Western blotting.

Protein Expression and Purification

The recombinant plasmids within the pET32a (+) vector were transformed into *Escherichia coli* BL21(DE3) competent cells. Single colonies were cultured in 5 mL of LB medium supplemented with 100 μg/mL ampicillin at 37 °C overnight, then transferred into 1 L of fresh LB medium with the same antibiotic and cultured at 37 °C until OD_{600} reached 0.6–0.8. Protein expression was induced by adding 0.8 mM IPTG (Sigma, I6758), and cells were incubated at 16°C for 16 h. Cell pellets were harvested by centrifugation and resuspended in ice-cold lysis buffer (50 mM Tris-HCl, pH 8.0, 300 mM NaCl, 20 mM imidazole) supplemented with protease inhibitor cocktail (MedChemExpress, Cat. No. HY-K0010). Cells were lysed by sonication, and crude lysates were clarified by centrifugation at 12,000 × g for 10 minutes at 4°C. The supernatant was loaded onto a Ni–NTA columns (Cytiva #17531801) pre-equilibrated with lysis buffer. After washed with lysis buffer, bound proteins were eluted with elution buffer (50 mM Tris-HCl, pH 8.0, 300 mM NaCl, 250 mM imidazole). Fractions containing target protein were pooled, concentrated, and analyzed by SDS-PAGE stained with Coomassie Brilliant Blue to assess purity and stability (SI Appendix, Fig. S13).

Surface Plasmon Resonance (SPR) analysis

Biacore™ T200 system (Cytiva, Uppsala, Sweden) was used to analyze the interaction between different proteins following the manufacturer’s introductions. Briefly, the ligand protein in 10 mM

sodium acetate buffer pH 4.5 (Cytiva, BR100350) were immobilized on the surface of a CM5 sensor chip (BR100012, Cytiva) using Amine Coupling Kit (Cytiva, BR100050) at a flow rate of 10 μL/min, blocked with ethanolamine (1 M, pH 8.5), and finally reached a resonance unit (RU) of ~3,500. Serial indicated concentrations of analyte proteins in HBS-EP+ buffer (0.1 M, HEPES; 1.5 M NaCl; 30 mM EDTA; 0.5% Surfactant P20; pH 7.4) were applied to analyze their interactions with immobilized VHSV-G or LjCD9 protein at a flow rate of 30 μL/min. VHSV (10^7 pfu/mL) in PBS at specified concentrations was also analyzed for interaction with immobilized LjCD9 protein. After each binding cycle, the chip surface was regenerated with 10 mM Glycine-HCl (pH 2.5) at 30 μL/min for 30 s to remove bound analytes. Blank buffer injections served as negative controls. Binding kinetics were analyzed using the BIA evaluation program (GE, USA) with a 1:1 binding model. Rate constants (association constant Ka, dissociation constant Kd) and equilibrium dissociation constant (K_D=Kd/Ka) were determined, ensuring kinetic parameters met instrument specifications.

Immunofluorescence (IF) assays

To visualize subcellular protein localization, the cells were seeded onto suitable coverslips and fixed with paraformaldehyde to preserve cellular structures and antigenicity. Permeabilization of the cells was achieved using 0.15% Triton X-100 (Merck, 648466) for 10 min, followed by blocking with 5% skim milk for 1 h. The cells were then incubated with primary antibodies targeting the proteins overnight at 4°C. After thorough washing with PBS, the cells were then incubated with Alexa Fluor 555- or 488-conjugated secondary antibodies (1:1,000), and the nuclei were counterstained using the DNA-specific dye DAPI (Sigma, D9542). The target proteins were visualized under a fluorescence microscope (Leica SP8).

Viral infection

LJB cells in 12-well plates were infected with VHSV (MOI=1) for 2, 4, 24, or 48 h. Tissue and cell samples were washed with PBS and collected for RNA isolation and RT-qPCR analysis.

For the binding assay, LJB cells in 12-well plates were incubated with medium containing VHSV in the presence of NTZ or DMSO at 4°C for 2 h to allow for virus binding without internalization. Unbound virus particles were removed by washing the cells with PBS three times and replacing the medium with fresh medium. The cells were cultured at 28°C for another 22 h, after which the viral gene expression in the cells was subsequently analyzed by RT-qPCR.

For the internalization assay, LJB cells were incubated with VHSV at 4°C for 2 h. After the unbound virus was removed by washing three times with PBS, the cells were incubated with NTZ or DMSO at 28°C for 2 h to promote virus internalization. The cells were then washed with PBS to eliminate the compounds and subsequently cultured at 28°C for another 20 h. Subsequent analysis was conducted using RT-qPCR.

Virus titration

Viral titers were determined by plaque assay. Vero cells were inoculated in a 12-well well plate. The samples were prepared in serial 10-fold dilutions, and 100 μL/ well of the diluted virus were added. The cells were incubated with the diluted virus at 37 °C for 1 h. Then the medium was removed and cultured in 2% methylcellulose (1:1) (Sigma-Aldrich) or the mixture of 2×MEM (Invitrogen) and isopycnic 2% low melting point agarose (Sangon Biotech) (1:1). Visible plaques were counted at 1 d 10% formaldehyde was used to fix cells and plaques were visualized by staining with 1% crystal violet.

Viral labeling and detection

The VHSV particles were labeled with DID (Invitrogen, USA) following the manufacturer's guidelines. The purified VHSV particles were gently shaken with 10 mM DID and incubated for 2 h in the dark at room temperature. Unincorporated DID was eliminated by exchanging the buffer with PBS using an NAP-10 filtration column (GE Healthcare, USA). The concentration of the labeled VHSV particles was assessed in FHM cells, and the movement of VHSV-DID was observed under a fluorescence microscope (Leica SP8).

Blocking assays

LJB cells in 24-well plates were incubated with anti-CD9 antibody (1:50) (Abcam) for 3 h at 28°C. After being washed with fresh media, the cells were infected with VHSV at 4°C for 2 or 4 h to allow binding without internalization, respectively. Rabbit IgG served as a negative control. The cells were then washed three times with PBS to remove unbound virus and harvested for RT-qPCR detection. VHSVs were incubated with different concentrations (100 or 500 ng) of affinity-purified His-LjCD9 proteins for 4 h at 4°C. His-tag control protein (expressed from empty pET-32a vector) (500 ng) was used as a negative control. Then, the LJB cells were incubated with virus and protein mixtures for 4 h at 4°C to facilitate binding and harvested for RT-qPCR detection.

Crystal Violet Staining

Confluent monolayer cells cultured in 24-well plates were infected with virus for 24 h. After viral infection, the culture medium was carefully removed and cells were fixed with 4% formaldehyde in PBS for 15 minutes at room temperature. Following three washes with PBS, cells were stained with 0.1% crystal violet (MedChemExpress, HY-B0324A) solution in 20% ethanol for 30 minutes. Excess stain was removed by rinsing cells with distilled water until the background was clear. After air-drying, the stained cells were visualized and photographed under a microscope.

Hematoxylin and Eosin (HE) Staining

Fish tissues from virus-infected and mock-infected control groups were collected and immediately fixed in 10% formalin (Sigma, 15512) at 4°C for 24 h. Fixed tissues were then dehydrated through a graded series of ethanol solutions (70%, 80%, 90%, and 100%) for 1 h each, followed by clearance in xylene for 2 h. Tissues were subsequently infiltrated and embedded in paraffin wax at 65°C. Serial sections (5 μm thick) were cut using a microtome and mounted on glass slides. Sections were deparaffinized in xylene and rehydrated through graded ethanol to distilled water. For HE staining, sections were immersed in hematoxylin solution for 5 minutes to stain nuclei blue, then rinsed and treated with eosin solution for 2 minutes to counterstain cytoplasm and extracellular components pink. After differentiation and washing, sections were mounted with coverslips and photographed under a light microscope.

Cell viability assay

The 50% cytotoxicity (CC_{50}) and the median effective concentration (EC_{50}) of NTZ and inhibitors were determined by cell viability assay using Cell Counting Kit-8 reagent (CCK-8, MCE, Cat #HY-K0301). FHM cells in 96-well plates was incubated with 100 μL fresh medium (2% FBS) containing serial diluted compound (NTZ: 1, 2, 4, 8, 16, 32, 64, 128 or 256 μM) in triplicate wells for 48 h at 28°C. After washed with PBS, the fresh medium (2% FBS) with CCK-8 solution (10 μL) was added to each well for 2 h at 28°C. Optical density (OD) was measured, and relative cell viability was calculated using formula: (OD_{450} [compounds-treated cells] - OD_{450} [medium]) / (OD_{450} [DMSO-treated cells] - OD_{450} [medium]) $\times$ 100%. The CC_{50} of NTZ and inhibitors was computed out in Graphpad Prism 8.0.2 (San Diego, California USA). For EC_{50} calculation, the cells were treated as above, and infected with VHSV for 48 h at 28°C. The EC_{50} of NTZ were computed out using [Agonist] vs. response -Variable slope (four parameters) algorithms in Graphpad Prism 8.0.2.

Inhibitor assays

LJB cells in 6-well plates were pre-treated with endocytosis inhibitors for 3 h at 28°C. The inhibitors included: CME inhibitors [CPZ (1.25 μg/mL), Dynasore (10 μg/mL)], CavME inhibitors [MβCD (5 μg/mL), Nystatin (20 μg/mL)], and the micropinocytosis inhibitor Wortmannin (1 μg/mL). Cells treated with 0.1% DMSO served as the control. After pre-treatment, cells were infected with VHSV (MOI=1) at 28°C for 4 h. Unbound viruses were removed by three washes with ice-cold PBS, and cells were further incubated in fresh medium at 28°C for 20 h. Total RNA was extracted using TRIzol reagent (Invitrogen) for RT-qPCR analysis.

Transmission electron microscopy (TEM)

For TEM analysis of VHSV virions in cell samples, the cells were collected and fixed at 4°C for 24 h in 2.5% glutaraldehyde in 0.1 M PBS (pH 7.4) or 2% osmium tetroxide in 0.1 M PBS. The ultrathin

sections were observed using a Hitachi SU8010 transmission electron microscope (Tokyo, Japan) as previously described (14).

Molecular AutoDocking

The 3D structure of the VSV G was retrieved from the Protein Data Bank (PDB ID: 5I2M) (15), the structure of the VHSV and SCRV G were predicted using the AlphaFold3 online server (https://alphafoldserver.com) (16). The chemical structure of the small molecule NTZ was retrieved from the PubChem database (CID: 446350). Molecular docking was performed using the CB-Dock2 platform (https://cadd.labshare.cn/cb-dock2) in Auto Blind Docking mode (17). The platform automatically identified potential binding pockets on the target proteins and evaluated ligand–protein interactions based on Vina scores, generating multiple binding conformations. The top-ranked binding pose was then visualized and analyzed using PyMOL v3.0 (Schrödinger, LLC).

Virus challenge

Healthy sea perch with a body length of 5-7 cm and an average body weight of 10 ± 2 g were acclimatized for 7 d and randomly divided into 4 groups (n=30 per group) before the experiment. All experiments were approved by the Institutional Animal Care and Use Committee of Sun Yat–sen University (SYSU-IACUC-2023-B0132). Fish were monitored every 12 h for mortality and morbidity symptoms, and survival rates were analyzed using GraphPad Prism 8.0.2 with the log-rank test.

Healthy sea perch fishes were intramuscularly injected with 200 μL of VHSV (3×10^7 $TCID_{50}$/ml) or PBS as a negative control. After 24 h, tissues, including heart, intestine, muscle, gill, liver, brain, kidney, and spleen, were collected from both groups.

For antibody blocking assay *in vivo*, fish were intraperitoneally injected with 30 μL of PBS containing 10 μg anti-CD9 antibody or PBS alone. After 4 h, all fish were challenged with VHSV (5.2×10^3 $TCID_{50}$/mL) or SCRV (4.8×10^3 $TCID_{50}$/mL) as previously reported (18). Negative control fish received an equal volume of PBS.

For protein blocking assay *in vivo*, VHSV was preincubated with purified His-LjCD9 recombinant protein or His-tagged protein at 4°C for 4 h. The mixtures were then intraperitoneally injected into separate fish groups; the negative control group received PBS.

For NTZ test *in vivo*, fish was intraperitoneally injected with 50 μL of a mixture containing VHSV and DMSO or NTZ (8 μM). A negative control group was injected with an equal volume of PBS.

Generation of CD9-deficient zebrafish

The AB strain wild-type zebrafish and CD9-deficient zebrafish lines (CZ2053) were purchased from the China Zebrafish Resource Center (http://zfish.cn/). The zCD9 gene (ENSDART00000135587.2) Exon 3 was precisely targeted for disruption employing the CRISPR/Cas9 genome editing platform.

A specific guide RNA (gRNA) was computationally designed against the zCD9 first exon sequence utilizing the ZiFiT online design tool (http://zifit.partners.org/ZiFiT), with the selected gRNA sequence (GGCAGCTCCAGGAACACT) positioned immediately upstream of an NGG Protospacer Adjacent Motif. The gRNA was enzymatically synthesized through *in vitro* transcription using PCR-amplified templates generated from the pMD19T-gRNA plasmid, with custom primers incorporating both target-specific and plasmid-adapted sequences (gRNA1-Forward primer (5'-3'): gatcccaagaccaagagttt; gRNA1-Reverse primer (5'-3'): ggggtctaataattcaggagag). Transcription was performed using the TranscriptAid T7 High-Yield Transcription Kit (K0441, Thermo Fisher Scientific) according to the manufacturer's protocol. For microinjection, the purified gRNA was combined with recombinant Cas9 nuclease (E365, Novoprotein) in injection buffer, with final working concentrations optimized to 200 ng/μL for Cas9 and 100 ng/μL for gRNA. The ribonucleoprotein complex was freshly prepared and microinjected into zebrafish embryos at the single-cell stage.

For viral challenge in zebrafish, three-month-old wild-type (WT) and CD9-knockout ($zCD9^{-/-}$) zebrafish, with an average length of 3.5 ± 0.3 cm and a body weight of 0.4 ± 0.05 g, were acclimated to 15°C by gradually decreasing the temperature for 7 d prior to infection. Fish were randomly assigned to three groups (n = 30 per group). The control group was intraperitoneally injected with 5 μL of sterile PBS. The experimental groups were intraperitoneally injected with 5 μL of VHSV at a dose of 5×10^6 $TCID_{50}$/mL. Following injection, fish were maintained at 15°C, and cumulative mortality was recorded daily, and internal organs (brain, intestine, liver or eyes) were collected for further pathological analysis. For molecular analysis, tissues were immediately snap-frozen in liquid nitrogen and stored at −80 °C until subsequent RNA extraction.

Generation of hCD9-knockout A549 cells by CRISPR/Cas9 gene editing

Oligonucleotide sequences of single guide RNA targeting human CD9 are 5'F: CACCGGCTGAAAGCCATCCACTATG; 3'R: AAACCATAGTGGATGGCTTTCAGCC. The pair of forward and reverse oligonucleotides for generation of sgRNA were annealed and inserted into plasmid vector lentiCRISPR v2 (Addgene, #52961) between BsmBI restriction sites. The sgRNA vector (pLenti-sCD9#1) and two packaging plasmids, pSPAX2 (Addgene, #12260) and pVSVG (Addgene, #12259), were transfected into HEK293T cells using Lipofectamine® 2000 reagent (Thermo). Lentivirus supernatants were collected at 48 h post transfection and transduced into A549 cells. One day later, cells were transferred to new cell culture plates and selected by puromycin (1 μg/mL) for more than 7 d to isolate single-cell clones. PCR amplification of the hCD9 target locus followed by DNA sequencing confirmed successful CRISPR-Cas9-mediated disruption (Table S1).

Data analysis

The data are shown as the mean ± SEM. Statistical significance was determined by Student's t test for two-group comparisons or one-way ANOVA for multiple-group comparisons. $p<0.05$ was considered to indicate statistical significance. $p<0.01$ was considered highly significant.

Declaration of interests

The authors declare no competing interests.

References

1. Y. Le *et al.*, Establishment and characterization of a brain cell line from sea perch, *Lateolabrax japonicus*. *In Vitro Cell Dev Biol Anim* **53**, 834-840 (2017).
2. N. Fijan *et al.*, Some properties of the Epithelioma papulosum cyprini (EPC) cell line from carp cyprinus carpio. **134**, 207-220 (1983).
3. M. Gravell, R. G. J. A. o. t. N. Y. A. o. S. Malsberger, A permanent cell line from fathead minnow (Pimephales promelas). **126**, 555-565 (1965).
4. C. Dong *et al.*, Development of a mandarin fish Siniperca chuatsi fry cell line suitable for the study of infectious spleen and kidney necrosis virus (ISKNV). *Virus Res.* **135**, 273-281 (2008).
5. D. J. Giard *et al.*, In vitro cultivation of human tumors: establishment of cell lines derived from a series of solid tumors. *J Natl Cancer Inst* **51**, 1417-1423 (1973).
6. F. L. Graham, J. Smiley, W. C. Russell, R. Nairn, Characteristics of a human cell line transformed by DNA from human adenovirus type 5. *J Gen Virol* **36**, 59-74 (1977).
7. N. C. Ammerman, M. Beier-Sexton, A. F. Azad, Growth and maintenance of Vero cell lines. *Curr Protoc Microbiol* **Appendix 4**, Appendix 4E (2008).
8. W. Zhang *et al.*, Isolation and identification of a viral haemorrhagic septicaemia virus (VHSV) isolate from wild largemouth bass *Micropterus salmoides* in China. *J Fish Dis* **42**, 1563-1572 (2019).
9. J. He *et al.*, Identification and functional analysis of the Mandarin fish (Siniperca chuatsi) hypoxia-inducible factor-1α involved in the immune response. *Fish Shellfish Immunol* **92**, 141-150 (2019).
10. M. H. Ng *et al.*, MIP-T3 is a negative regulator of innate type I IFN response. *J Immunol* **187**, 6473-6482 (2011).
11. M. A. Whitt, Generation of VSV pseudotypes using recombinant ΔG-VSV for studies on virus entry, identification of entry inhibitors, and immune responses to vaccines. *J. Virol. Methods* **169**, 365-374 (2010).
12. X. Lu *et al.*, N Protein of Viral Hemorrhagic Septicemia Virus Suppresses STAT1-Mediated MHC Class II Transcription to Impair Antigen Presentation in Sea Perch, Lateolabrax japonicus. *J Immunol* **208**, 1076-1084 (2022).
13. W. Zhang *et al.*, Ring Finger Protein 34 Facilitates Nervous Necrosis Virus Evasion of Antiviral Innate Immunity by Targeting TBK1 and IRF3 for Ubiquitination and Degradation in Teleost Fish. *J. Virol.* **97**, e0053323 (2023).
14. W. Zhang *et al.*, Marine medaka heat shock protein 90ab1 is a receptor for red-spotted grouper nervous necrosis virus and promotes virus internalization through clathrin-mediated endocytosis. *PLoS Pathog* **16**, e1008668 (2020).
15. S. Roche, S. Bressanelli, F. A. Rey, Y. Gaudin, Crystal structure of the low-pH form of the vesicular stomatitis virus glycoprotein G. *Science* **313**, 187-191 (2006).
16. J. Abramson *et al.*, Accurate structure prediction of biomolecular interactions with AlphaFold 3. *Nature* **630**, 493-500 (2024).
17. Y. Liu *et al.*, CB-Dock2: improved protein-ligand blind docking by integrating cavity detection, docking and homologous template fitting. *Nucleic Acids Res* **50**, W159-w164 (2022).
18. N. Lorenzen, N. J. Olesen, P. E. Jørgensen, Neutralization of Egtved virus pathogenicity to cell cultures and fish by monoclonal antibodies to the viral G protein. *J Gen Virol* **71 (Pt 3)**, 561-567 (1990).

Figures

Fig. S1.

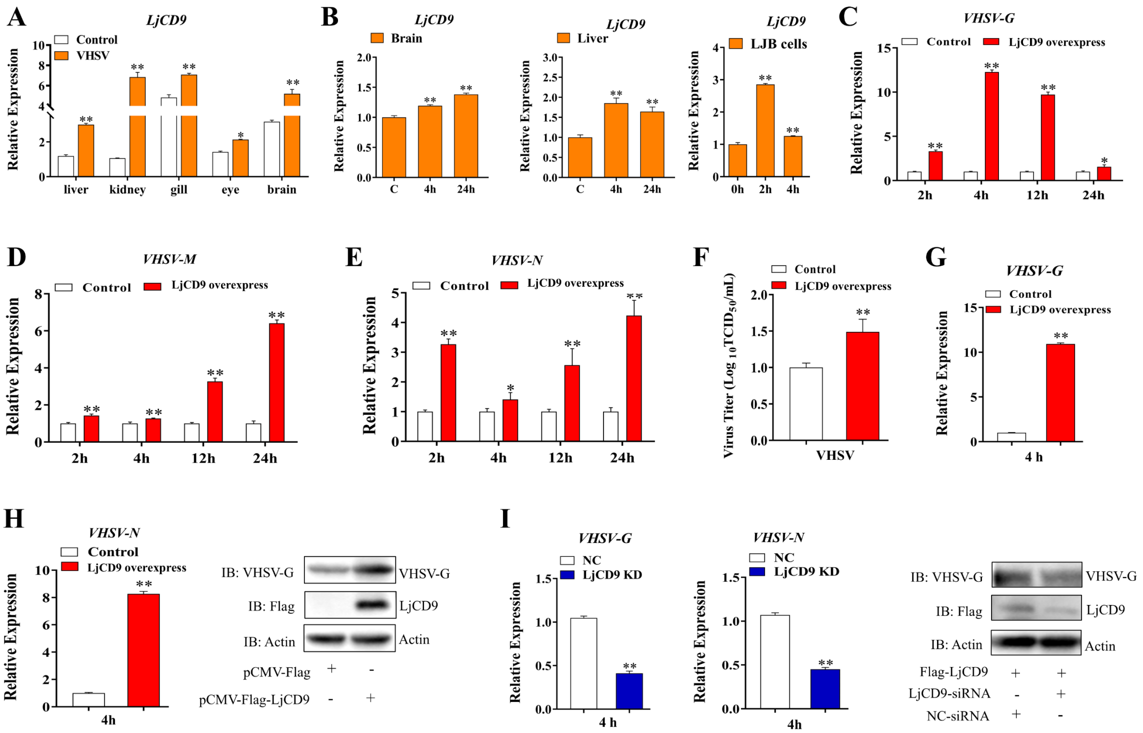

***SI Appendix*, Fig. S1. LjCD9 is associated with VHSV infection. (A)** RT-qPCR analysis of *LjCD9* mRNA expression in VHSV-infected sea perch tissues at 24 hpi. **(B)** Temporal expression profiles of *LjCD9* mRNA in the brain and liver tissues, as well as LJB cells following VHSV infection, compared to the PBS-injected control group. **(C-E)** RT-qPCR analysis of VHSV *G*, *M*, and *N* mRNA in LjCD9-overexpressing LJB cells following inoculation with VHSV, relative to Flag-tag transfected control cells at 2, 4, 12 and 24 h. **(F)** Viral titers in supernatants of LjCD9-overexpressing LJB cells post-VHSV infection for 24 h. **, $p < 0.01$. **(G-H)** Effect of LjCD9 overexpression on VHSV infection. RT-qPCR analysis of VHSV *G* and *N* gene in LjCD9-overexpressed LJB cells post VHSV infection for 4 h; the VHSV G protein expression was further analyzed by Western blot following 20 h incubation. **(I)** Effect of LjCD9 knockdown on VHSV infection. RT-qPCR analysis of VHSV *G* and *N* genes in LJB cells transfected with LjCD9 siRNA and infected with VHSV for 4 h. The VHSV G protein expression was further analyzed by Western blot following 20 h incubation. Results are representative of three independent experiments. Data are means ± SEM. ** $p < 0.01$.

Fig. S2.

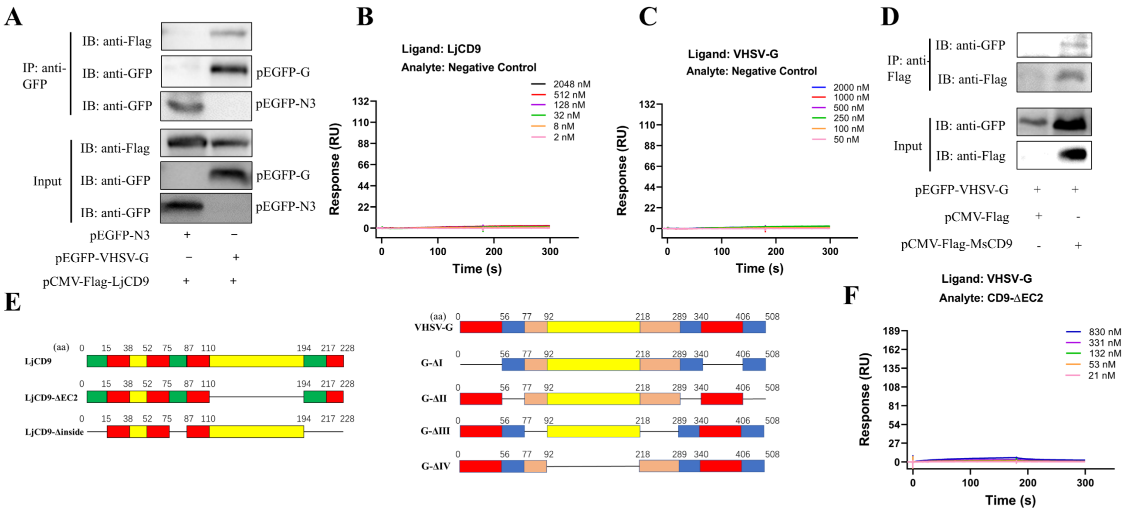

***SI Appendix*, Fig. S2. LjCD9 interacted with VHSV G protein. (A)** Co-IP of LjCD9 and VHSV G protein. HEK293T cells were transfected with pEGFP-N3 or pEGFP-VHSV-G with pCMV-Flag-LjCD9. The cell lysates were then subjected to immunoprecipitation using anti-GFP magnetic beads followed by western blot analysis. **(B-C)** SPR negative control assays. His-tagged protein was passed over immobilized LjCD9 (B) or VHSV G (C) on a CM5 sensor chip to confirm non-specific binding. **(D)** Co-IP of VHSV G with MsCD9 (*Micropterus salmoides* CD9). Lysates from HEK293T cells transfected with Flag-MsCD9 and pEGFP-VHSV-G plasmids were subjected to immunoprecipitation with anti-Flag antibody. **(E)** Schematic diagrams of LjCD9 and VHSV G truncation mutants. **(F)** SPR analysis of LjCD9-ΔEC2 mutant binding to immobilized VHSV G. VHSV G protein was coupled to a CM5 chip, and LjCD9-ΔEC2 protein was passed at the indicated concentrations. Data are representative of three independent experiments.

Fig. S3.

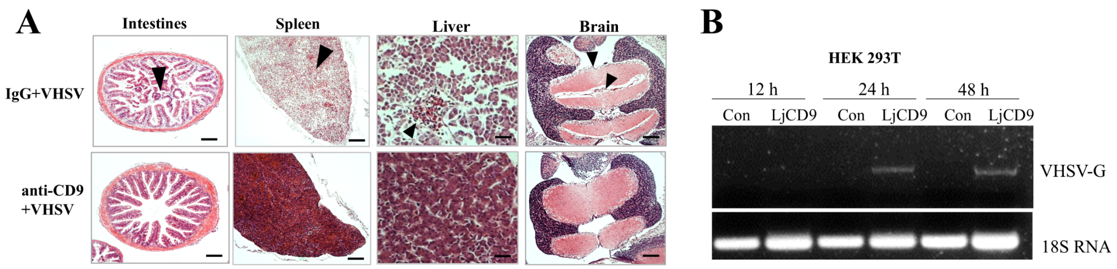

***SI Appendix*, Fig. S3. LjCD9 promotes VHSV infection. (A)** Effect of anti-CD9 antibody on VHSV infection *in vivo*. Histopathology of sea perch with an intraperitoneal injection of 10 μg anti-CD9 antibody in 30 μL of PBS, or PBS alone for 3 h, following injection with VHSV ($TCID_{50}=5.2\times10^{3}$). Negative control received PBS only, **, $p<0.01$. The intestine, spleen, liver and brain sections from sea perch were subjected to histopathology observation, Bar=100 μm. **(B)** RT-PCR detection of VHSV G bands in LjCD9-overexpressing HEK293T cells post-VHSV infection, washed to remove unbound virus, and incubated for 12, 24 or 48 h, respectively.

Fig. S4.

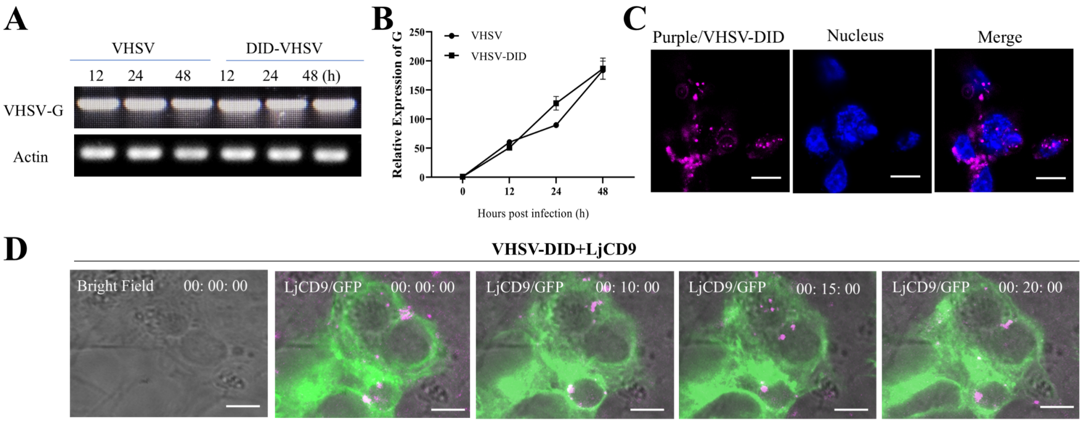

***SI Appendix*, Fig. S4. Characterization of DID-labeled VHSV.** **(A)** RT-PCR detection of VHSV-*G* mRNA expression in VHSV and VHSV-DID infected LJB cells at 12, 24 and 48 hpi. **(B)** RT-qPCR analysis of VHSV *G* mRNA in VHSV and VHSV-DID infected LJB cells at 12, 24 and 48 hpi. **(C)** Fluorescence microscopy of DID-labeled VHSV (purple) in infected LJB cells; nuclei stained with DAPI (blue). Bar=10 μm. **(D)** Bright filed and merged images of HEK293T cells with colocalization of DID-labeled VHSV virions (purple) with GFP-LjCD9 (green) in cells after 4 h incubation (28°C). Bar=10 μm.

Fig. S5.

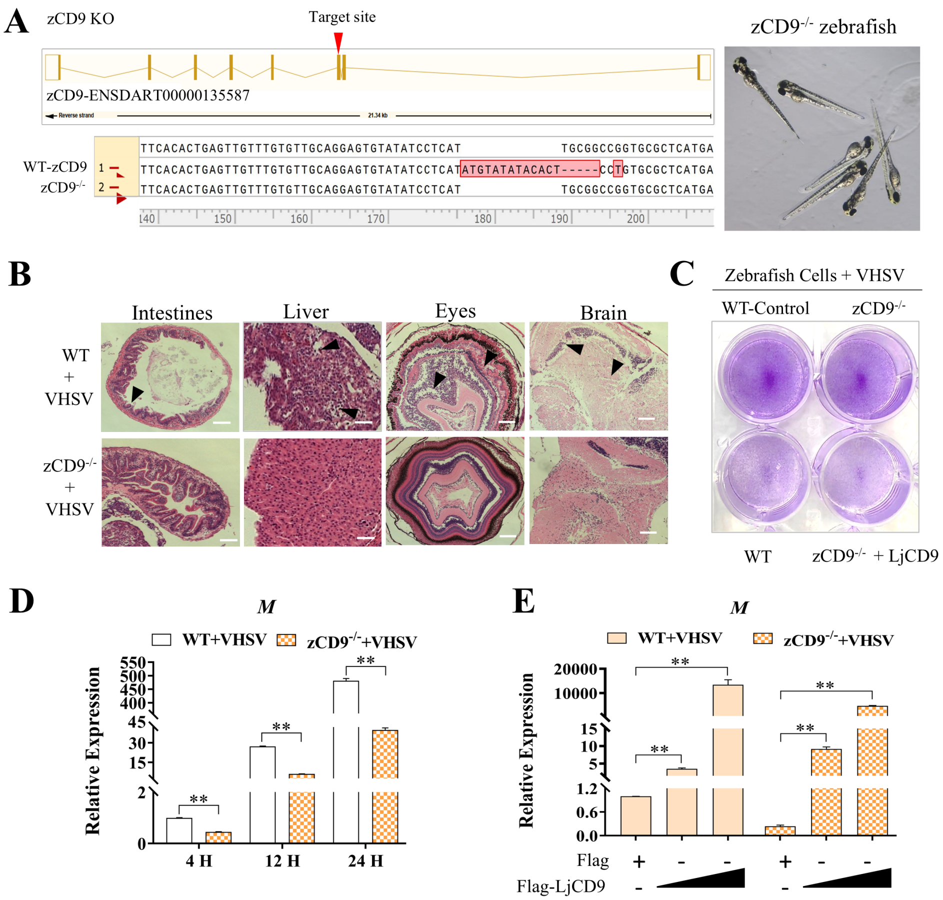

***SI Appendix*, Fig. S5. CD9 knockout greatly attenuates VHSV infection. (A)** CRISPR-Cas9 target sequence for zebrafish CD9 (zCD9) knockout. **(B)** Effect of zCD9 knock out on VHSV infection *in vivo*. Histopathology of wild-type (WT) or CD9 knock out ($CD9^{-/-}$) zebrafish intraperitoneally infected with VHSV at 15˚C. PBS-injected fish as control. The intestine, liver, eyes and brain from VHSV-infected WT and $zCD9^{-/-}$ zebrafish were subjected to histopathology observation, Bar=100 μm. **(C)** Plaque staining of VHSV-infected primary cells derived from WT or $zCD9^{-/-}$ zebrafish at 24 hpi. **(D)** VHSV *M* mRNA levels in WT and $zCD9^{-/-}$ zebrafish cells at 4, 12, and 24 hpi. **, $p < 0.01$. **(E)** VHSV *M* mRNA levels in $zCD9^{-/-}$ zebrafish cells rescued with Flag-LjCD9 or empty vector, followed by VHSV infection for 24 h. **, $p < 0.01$.

Fig. S6.

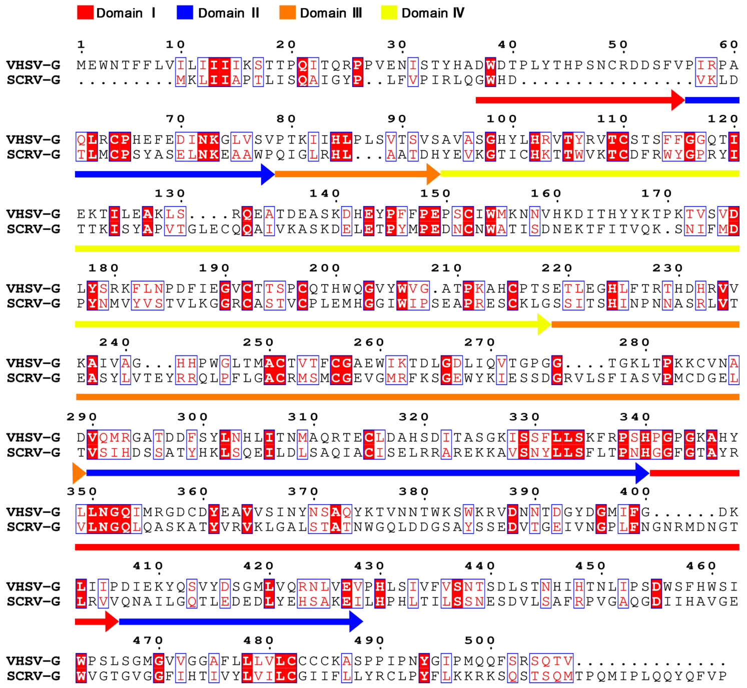

***SI Appendix*, Fig. S6. G protein sequence alignment of VHSV and SCRV.** The G proteins of VHSV and SCRV were divided into four domains and labeled in different colors. Sequence alignment of these G proteins was performed using MEGA-X and visualized with ENDscript 2, enabling comparative analysis of their molecular features and potential functional regions.

Fig. S7.

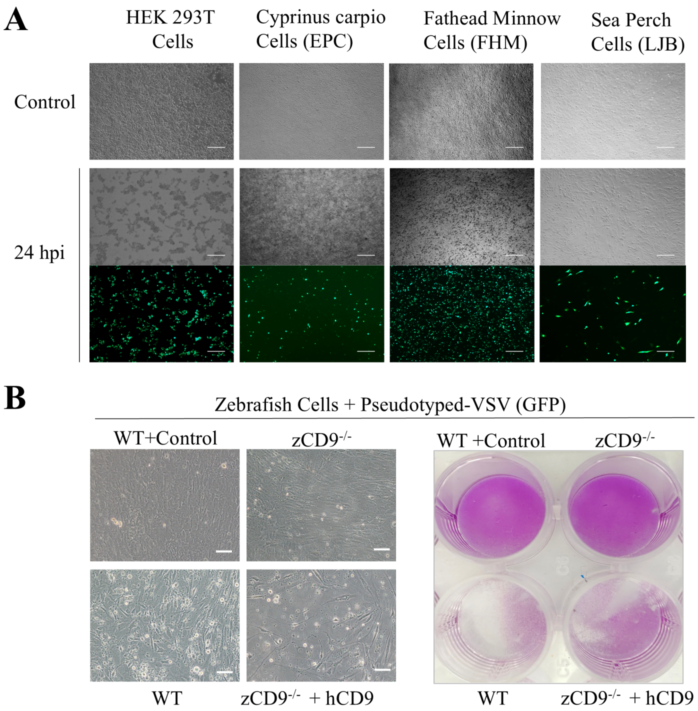

***SI Appendix*, Fig. S7. Viral susceptibility of fish cells to VSV infection. (A)** Cytopathic effects and fluorescence images of different fish cells (EPC, FHM and LJB) infected with pseudotyped-VSV (GFP) for 24 h. HEK293T cells were infected as positive control, respectively. Bar=10 μm. **(B)** Cytopathic effects and plaque staining of WT, $CD9^{-/-}$ zebrafish cells or $CD9^{-/-}$ zebrafish cells with hCD9 overexpression, followed by pseudotyped-VSV (GFP) infection for 24 h. Bar=10 μm.

Fig. S8.

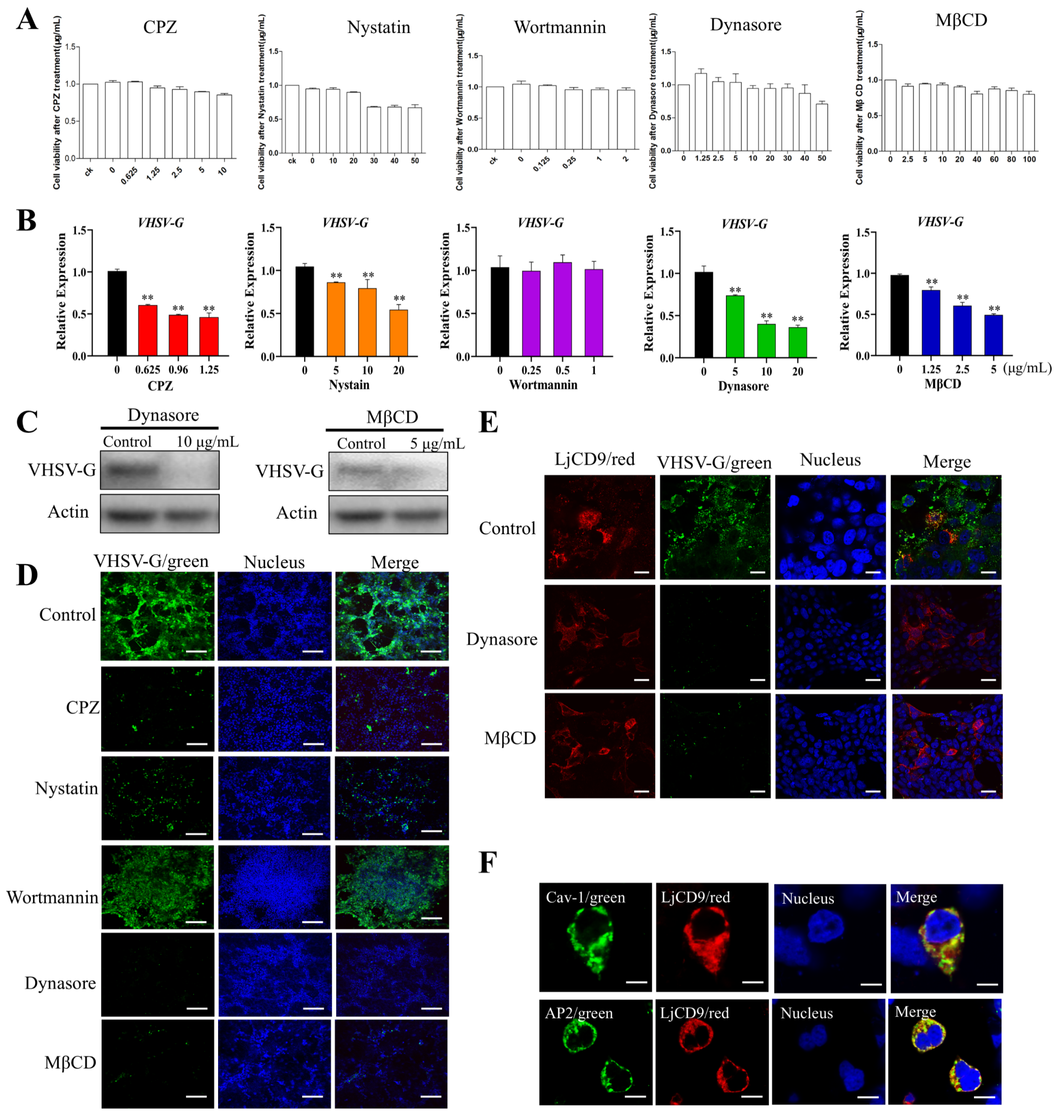

***SI Appendix*, Fig. S8. VHSV entry into LJB cells through clathrin-mediated and caveolae-mediated endocytosis. (A)** The cell viability of LJB cells post-treatment with the inhibitors at indicated concentrations**. (B-D)** Inhibition of VHSV entry by endocytic pathway blockers. LJB cells pretreated with clathrin inhibitors (chlorpromazine [CPZ] and Dynasore), caveolae inhibitors (Nystatin and methyl-β-cyclodextrin [MβCD]), or micropinocytosis inhibitor (wortmannin) were infected with VHSV (4 h, inhibitors present), washed, and subjected to VHSV *G* gene expression by RT-qPCR (**B**), the VHSV G protein was further detected by immunoblotting (**C**) and immunofluorescence analysis (**D**) following 20 h incubation. **, $p < 0.01$; Bar=10 μm. **(E)** LjCD9 overexpression enhances endocytosis-dependent VHSV infection. Immunofluorescence analysis of VHSV entry in inhibitors-treated HEK293T cells. Flag-LjCD9-transfected cells pretreated with

inhibitors were infected with VHSV (4 h), washed, incubated 20 h, and stained for VHSV G (green; anti-VHSV G) and LjCD9 (red; anti-Flag) proteins. Bar=10 μm. **(F)** Confocal microscopy of HEK293T cells cotransfected with Flag-LjCD9 and Myc-Cav1 or Myc-AP2 plasmids for 24 h, stained with anti-Flag (LjCD9, red) and anti-Myc (Cav1/AP2, green) antibodies. Bar=10 μm.

Fig. S9.

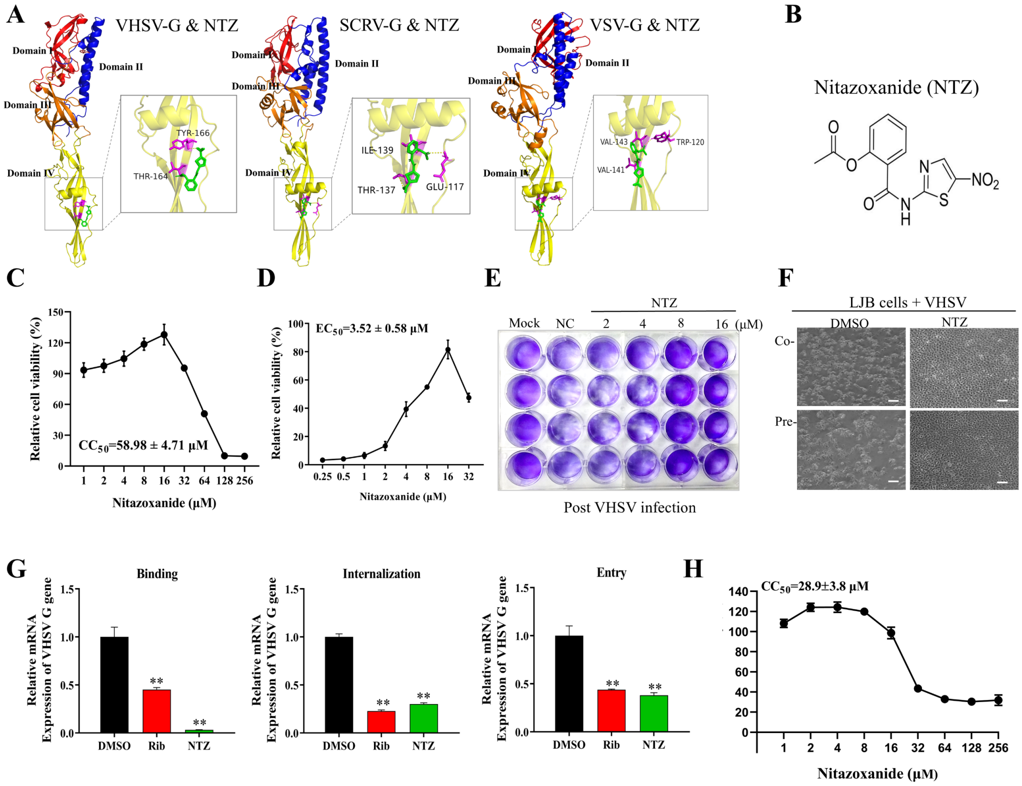

***SI Appendix*, Fig. S9. Nitazoxanide targets viral G protein to block viral entry. (A)** Molecular docking analysis of NTZ binding to VHSV G, SCRV G, and VSV G. AutoDock Vina was used to predict interaction modes; left panels show overall binding conformations, and right panels highlight interacting domains and key residues. **(B)** Structural formula of NTZ. **(C)** Cytotoxicity assay of NTZ in LJB cells. CC_{50} was determined by treating cells with serial NTZ concentrations for 48 h followed by cell viability assessment. **(D)** Antiviral efficacy assay of NTZ against VHSV. EC_{50} was calculated by infecting LJB cells with VHSV ($TCID_{50}$=5.2×10^3), treating with NTZ, and measuring viability at 48 hpi. **(E)** NTZ inhibits VHSV infection in a dose-dependent manner. LJB cells were infected with VHSV and treated with different concentrations of NTZ for 36 h, followed by plaque staining. **(F)** Cytopathic effects of LJB cells treated with NTZ and VHSV (co-exposure), or pre-incubated with NTZ for 3 h, followed by VHSV infection for 36 h (pre-incubation). DMSO were used as negative control, respectively. Bar=50 μm. **(G)** Kinetics of NTZ inhibition on VHSV binding, internalization, and entry. LJB cells were treated with NTZ, ribavirin (Rib, positive control), or DMSO at different time points post-infection, and VHSV *G* expression was analyzed by RT-qPCR. **, $p<0.01$. **(H)** Cytotoxicity assay of NTZ in HEK 293T cells. CC_{50} was determined by treating cells with serial NTZ concentrations for 48 h followed by cell viability assessment.

Fig. S10.

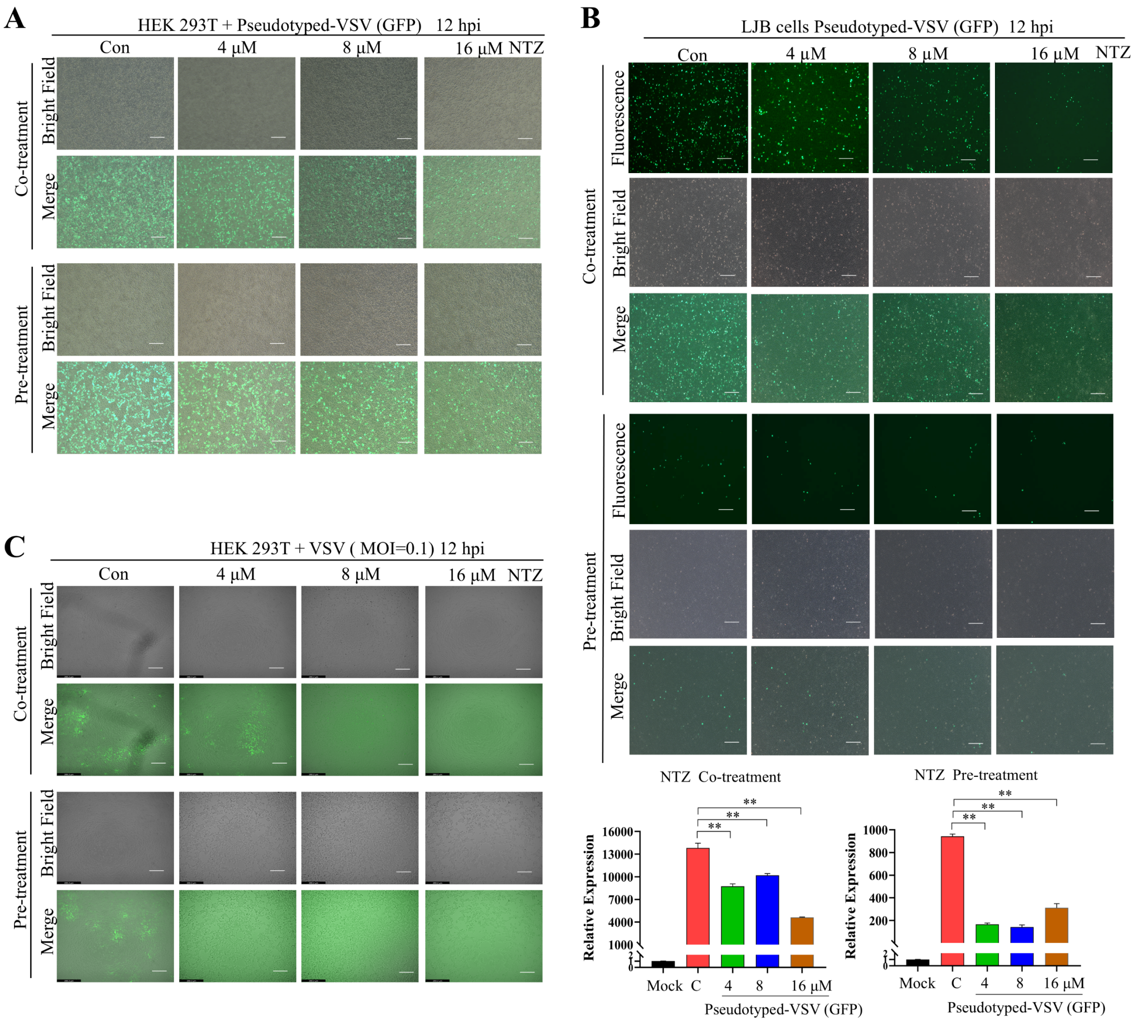

***SI Appendix*, Fig. S10. Inhibitory effects of NTZ on VSV infection in HEK293T cells. (A)** The bright field and merge images of HEK293T cells, that were infected by pseudotyped-VSV (GFP) with co-treating with dose-dependent NTZ or pre-incubating with dose-dependent NTZ for 3 h, then followed by incubation for 12 h. Ba=200 μm. **(B)** Inhibitory effects of NTZ on VSV infection in LJB cells. LJB cells were infected by pseudotyped-VSV (GFP) with co-treating with dose-dependent NTZ or pre-incubating with dose-dependent NTZ for 3 h, followed by RT-qPCR at 12 hpi. Fluorescence, bright field and merge images were captured at 12 hpi. **, $p < 0.01$. Bar=200 μm. **(C)** The bright field and merge images of HEK293T cells, that were infected by wild-type VSV, with co-treating with dose-dependent NTZ or pre-incubating with dose-dependent NTZ for 3 h, then followed by incubation for 12 h. Bar=200 μm.

Fig. S11.

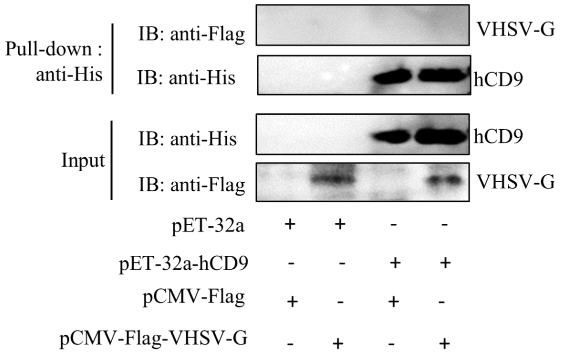

***SI Appendix*, Fig. S11. VHSV G did not interact with hCD9.** The lysates of HEK293T cells transfected with indicated plasmids were pulled down with purified His-hCD9 or His proteins using anti-His magnetic beads, and immunoblotted with anti-His and anti-Flag antibodies.

Fig. S12.

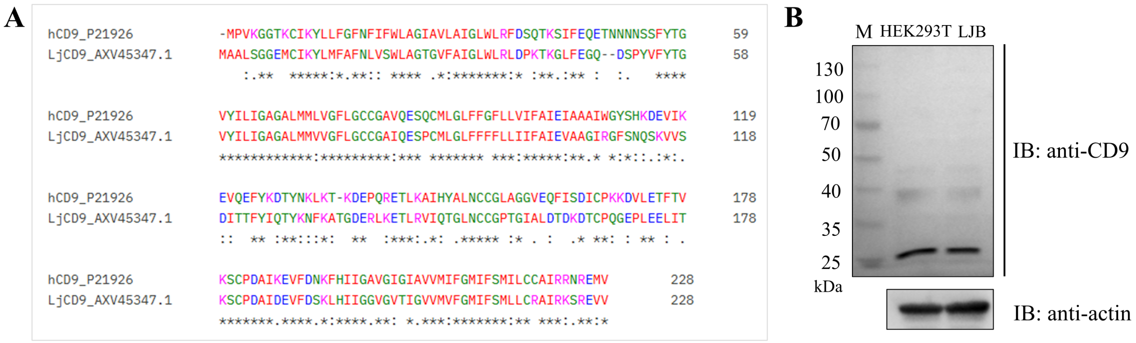

***SI Appendix*, Fig. S12. The specificity and sensitivity of anti-CD9 antibodies.** (**A**) Sequence alignment of hCD9 and LjCD9 protein. (**B**) Western blotting analysis of CD9 in HEK293T and LJB cells. Nitrocellulose membranes were incubated with anti-CD9 and anti-actin antibodies.

Fig. S13.

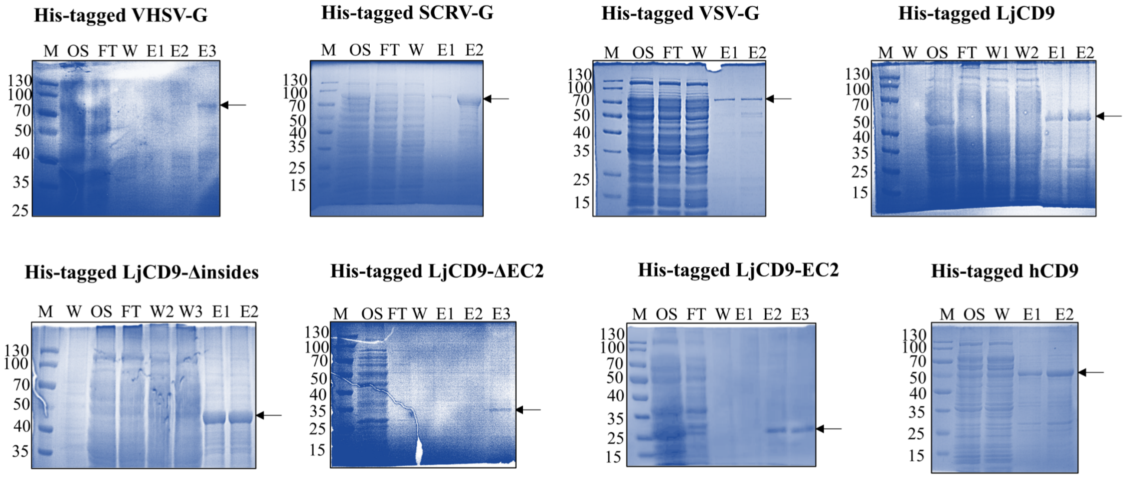

SI Appendix, Fig. S13. Purification of His-tagged proteins by Ni-NTA affinity chromatography. His-tagged recombinant proteins, including VHSV-G, SCRV-G, VSV-G, LjCD9, LjCD9-Δinsides, LjCD9-ΔEC2, LjCD9-EC2, hCD9 were purified on Ni–NTA columns, eluted with imidazole, and examined with Coomassie Brilliant Blue analysis. M, Protein marker; OS, Starting material (crude protein solution); FT, Flow-through; W, W1, W2, Wash fractions; E1–E3, Elution fractions.

Tables

Table S1. Sequence alignment of the CD9 knockout construct used in this study.

	Sequence	
	**WT**	**CD9 $^{-/-}$**
**zCD9**	CTTCACACTGAGTTGTTTGTGTTGCAG<br>GAGTGTATATCCTCATTGCGGCCGGT<br>GCGCTCATGATGGTGGTGGGATTTTT<br>CGGATGTTGCGGAGCCATCCAGGAG<br>TCCCCTTGCATGCTGGGATTGGT	CTTCACACTGAGTTGTTTGTGTTGCAGG<br>AGTGTATATCCTCATATGTATATACACT<br>CCTGTGCGCTCATGATGGTGGTGGGAT<br>TTTTCGGATGTTGCGGAGCCAGGAGCC<br>AGGAGTCCCCTTGCATGCTGGGATTGG<br>T
**hCD9**	CACCCCGTCCCCTCGTTGCCTTCCAG<br>TTCTTCGGCTTCCTCTTGGTGATATTC<br>GCCATTGAAATAGCTGCGGCCATCTG<br>GGGATATTCCCACAAGGATGAGGTAG<br>GTTTTTCCCATGAGATCTCTTGGGTTT<br>GGGAGTT	CACCCCGTCCCCTCGTTGCCTTCCAGT<br>TCTTCGGCTTCCTCTTGGTGATATTCGC<br>CATTGAAAT···2bp···CTGCGGCCATCTG<br>GGGATATTCCCACAAGGATGAGGTAGG<br>TTTTTCCCATGAGATCTCTTGGGTTTGG<br>GAGTT<br><br>CACCCCGTCCCCTCGTTGCCTTCCAGT<br>TCTTCGGCTTCCTCTTGGTGAT<br>ATTCGCCAT···18bp···CTGGGGATATTC<br>CCACAAGGATGAGGTAGGTTTTTCCCA<br>TGAGATCTCTTGGGTTTGGGAGTT<br><br>CACCCCGTCCCCTCGTTGCCTTCCAGT<br>TCTTCGGCTTCCTCTTGGTGAT<br>ATTC···39bp···AAGGATGAGGTAGGTTT<br>TTCCCATGAGATCTCTTGGGTTTGGGA<br>GTT

Red letters indicate the regions with sequence differences.

Table S2. Primer sequences of the genes used in this study.

Primer Name	primer 5’	primer 3’
Flag-VHSV-G	CGGAATTCCGATGGAATGGAATACTTTTTTCTT	CGGGGTACCTCAGACCATCTGGCTTCTG
VHSV-N-RT-qPCR	TTGGAGAACTGCAACACTTCAC	CGGTCAGGATGAAGGCGTAG
VHSV-G-RT-qPCR	CTATGACAGCGGAATGCTCGT	TCTGAAGGAATTAGATTGGTGTG
VHSV-M-RT-qPCR	CTATTCCAGAGTGCCATGATCT	TATGCGAGACCTCACTGGACT
VHSV-P-RT-qPCR	CAAGGACACCAGATCAGCTCT	TGGCATCCAGTTGAAGATCTTC
GFP-VHSV-G	GGGGTACCATGGAATGGAATACTTTTTTCTTGGT	GGGGTACCATGGAATGGAATACTTTTTTCTTGGT
Myc-VHSV-G-Δ Ⅰ（57-340 aa）	CTTATGGCCATGGAGGCCCGAATTCGGCCAGATCAACTCAG	GATGTCGTGACTAGGACGAAA
Myc-VHSV-G-Δ Ⅰ（407-507 aa）	AGTCACGACATCGAGAAATAC	GATCCCCGCGGCCGCGGTACCTAGACCATCTGGCTTCTGGAGA
Myc-VHSV-G-Δ Ⅱ（1-56 aa)	CTTATGGCCATGGAGGCCCGAGAATGGAATACTTTTTTCTT	TGGGACCGGAACAAAGGAGTC
Myc-VHSV-G-Δ Ⅱ（78-289 aa)	GTTCCGGTCCCAGCCCAGATC	CCCCGGGTCGGTGTTGACACA
Myc-VHSV-G-Δ Ⅱ（341-406 aa)	ACCGACCCGGGGCCGGGCAAG	GATCCCCGCGGCCGCGGTACCTATGGGATGATCAACTTGTCCC
Myc-VHSV-G-Δ Ⅲ（1-77 aa）	CTTATGGCCATGGAGGCCCGAGAATGGAATACTTTTTTCTT	TGCTGAAGAGACCAAGCCCTT
Myc-VHSV-G-Δ Ⅲ（93-218 aa）	GTCTCTTCAGCAGTCGCAAAT	CTGAACTGAGGTCGGGCAATG
Myc-VHSV-G-Δ Ⅲ（290-507 aa）	ACCTCAGTTCAGATGAGGGGA	GATCCCCGCGGCCGCGGTACCTAGACCATCTGGCTTCTGGAGA
Myc-VHSV-G-Ⅳ（1-92 aa）	CTTATGGCCATGGAGGCCCGAGAATGGAATACTTTTTTCTT	TGTTTCGACGCTGGTGACTGA
Myc-VHSV-G- Ⅳ（219-507 aa）	AGCGTCGAAACACTTGAGGGG	GATCCCCGCGGCCGCGGTACCTAGACCATCTGGCTTCTGGAGA
His-VHSV-G	CCCAAGCTTGGGAATGGAATACTTTTTTCTT	CCGCTCGAGGGACCATCTGGCTTCTGGAGA
Flag-LjCD9	CGGAATTCGGATGGCTGCGCTGTCGGGAG	CCGCTCGAGTTACACCACTTCCCGAGACTT
Flag-LjCD9-Δin	CTTATGGCCATGGAGGCCCGAGACCCAAAGACCAAAGGCCT	GATCCCCGCGGCCGCGGTACTTAGCACAGGAGCATGCTAAAGA
Flag-LjCD9-Δout	CTTATGGCCATGGAGGCCCGAGCTGCGCTGTCGGGAGGAGA	GATCCCCGCGGCCGCGGTACTTAAAAACCCCGGATTCCAG

His-LjCD9-Δin	CCCAAGCTTGGGACCCAAAGACC AAAGGCCT	CCGCTCGAGG TTA GCA CAG GAG CAT GCT AAA GA
His-LjCD9-Δout	CCCAAGCTTGG GCTGCGCTGTCGGGAGGAGA	CCGCTCGAGG TTA AA AAC CCC GGA TTC CAG
GST-LjCD9	TCCAGCTTCTGCAGGAATTCGCTG CGCTGTCGGGAGGAGA	AGCAGCCGGATCTCACTCGAGTTAC ACCACTTCCCGAGACTTCC
Flag-hCD9	CTTATGGCCATGGAGGCCCGACC GGTCAAAGGAGGCACCAA	GATCCCCGCGGCCGCGGTACCTAGA CCATCTCGCGGTTCC
His-hCD9	CTGATATCGGATCCGAATTCATGC CGGTCAAAGGAGGCAC	TCGAGTGCGGCCGCAAGCTTCTAGA CCATCTCGCGGTTCC
Flag-hCD9-Δout	CTTATGGCCATGGAGGCCCGACC GGTCAAAGGAGGCACCAA	GATCCCCGCGGCCGCGGTACCTATC CCCAGATGGCCGCAGCTAT
Flag-hCD9-Δin	CTTATGGCCATGGAGGCCCGACT GCTGTTCGGATTTAACTTC	GAT CCC CGC GGC CGC GGT AC CTA CAA GAT CAT ACT GAA GAT CAT
Flag-SCRV-G	CGGAATTCCGATGAAATTGATCAT TGCACTTAC	CGGGGTACCTTAGGGAACAAATTGA TACTGC
SCRV-M-RT-qPCR	CGGTTGCCATCTCTTATGA	CCTCTGCTTCTGCTATCTG
SCRV-N-RT-qPCR	TCGCATCATTCACTGGATT	TGGCAGAGTAAGGAGACA
SCRV-P-RT-qPCR	ACAGCAGAGGTCTCAAGA	ATTAGCATCCGCAGAAGG
VSV-N-RT-qPCR	AGCAAGGAATGCCCGACA	GCATTAGTCGTCAATCCTCCG
VSV-P-RT-qPCR	CCCAGTGGCTTTCGACGAT	TGATTGGGACGGATGTGTGTT
Flag-VSV-G	CGGAATTCCGATGGAATGGAATAC TTTTTTCTT	CGGGGTACCTCAGACCATCTGGCTT CTG
Myc-Cav1	CGGAATTCGGATGACAGGAGAAC TGAAGGACG	GGGGTACCCTACACCTCCTTGCTTG TGCG
Myc-AP2	CGGAATTCGGATGATCGGAGGAC TCTTCATCT	CCGCTCGAGTCAGCAGCGTGTTTCG TAGAT
β-Actin-RT-qPCR	GGAGCACCCAGTCCTGCTC	TGGTACGACCAGAGGCATACA
VHSV-G-RT-PCR	ATGGAATGGAATACTTTTTTC	TCAGACCATCTGGCTTCTGGA
β-Actin-RT-PCR	GTTGCATAATGAACTCGTGT	TAAGAATATCAGGCCTGCAAA
18sRNA-RT-PCR	TTCGATGGTAGTCGCCGTGCC	CTCCAATGGATCCTCGTTA

Table S3. GenBank accession numbers of genes and sequence information of mutants used in this study.

Gene Name	Accession Number	Sequence Information	
LjCD9	MH191344.1	Full length	1-687 bp
		LjCD9Δin	48-225 bp;261-582 bp
		LjCD9ΔEC2	1-330 bp;583-687 bp
		LjCD9EC2	331 bp-582 bp
hCD9	NM_001413243.1	Full length	1-687 bp
		hCD9Δin	37-243 bp;261-648 bp
		hCD9ΔEC2	1-330 bp;580-687 bp
		hCD9EC2	331-579 bp
Cav1	PX912443	Full length	1-546 bp
AP2	PX912444	Full length	1-937 bp
MsCD9	XM_038705298.1	Full length	684 bp
VHSV-G	MK598848.1	Full length	1524 bp
		VHSV-G-Δ Ⅰ	169-1020 bp;1219-1524 bp
		VHSV-G-Δ Ⅱ	1-168 bp;232-867 bp;1021-1218 bp;1285-1524 bp
		VHSV-G-ΔⅢ	1-231 bp;277-654 bp;868-1524 bp
		VHSV-G-ΔⅣ	1-276 bp;655-1524 bp
		VHSV-G-Ⅳ	277-654 bp
SCRV-G	PQ066877.1	Full length	1527 bp
VSV G	NC_001560.1	Full length	1533 bp
IHNV G	NC_001652.1	Full length	1527 bp
